# Exogenous miRNAs from *Moringa oleifera* Lam. recover a dysregulated lipid metabolism

**DOI:** 10.3389/fmolb.2022.1012359

**Published:** 2022-11-17

**Authors:** Valentina Roglia, Marina Potestà, Alessandra Minchella, Stefania Paola Bruno, Roberta Bernardini, Daniele Lettieri-Barbato, Federico Iacovelli, Angelo Gismondi, Katia Aquilano, Antonella Canini, Rosario Muleo, Vittorio Colizzi, Maurizio Mattei, Antonella Minutolo, Carla Montesano

**Affiliations:** ^1^ Department of Biology, University of Rome Tor Vergata, Rome, Italy; ^2^ UNESCO Interdisciplinary Chair in Biotechnology and Bioethics, Rome, Italy; ^3^ Bambino Gesù Children’s Hospital (IRCCS), Rome, Italy; ^4^ Department of Science, University Roma Tre, Rome, Italy; ^5^ Interdepartmental Center for Animal Technology, University of Rome Tor Vergata, Rome, Italy; ^6^ Santa Lucia Foundation IRCCS, Rome, Italy; ^7^ Department of Agricultural and Forestry Science, University of Tuscia, Viterbo, Italy; ^8^ Department of Experimental Medicine, University of Rome Tor Vergata, Rome, Italy

**Keywords:** plant miRNAs, *Moringa oleifera* Lam, cross-kingdom interactions, lipid metabolism, inflammatioin

## Abstract

A balanced diet is critical for human health, and edible plants play an important role in providing essential micronutrients as well as specific microRNAs (miRNAs) that can regulate human gene expression. Here we present the effects of *Moringa oleifera* (MO) miRNAs (*mol*-miRs) on lipid metabolism. Through *in silico* studies we identified the potential genes involved in lipid metabolism targeted by *mol*-miRs. To this end, we tested the efficacy of an aqueous extract of MO seeds (MOES), as suggested in traditional African ethnomedicine, or its purified miRNAs. The biological properties of MO preparations were investigated using a human derived hepatoma cell line (HepG2) as a model. MOES treatment decreased intracellular lipid accumulation and induced apoptosis in HepG2. In the same cell line, transfection with *mol*-miRs showed similar effects to MOES. Moreover, the effect of the *mo*l-miR pool was investigated in a pre-obese mouse model, in which treatment with *mol*-miRs was able to prevent dysregulation of lipid metabolism.

## 1 Introduction

Given the importance of nutrition to human health, ensuring access to good food for all and ensuring its quality and safety, is one of the most important challenges facing the international community (FAO, IFAD et al., 2021). Malnutrition has a major impact on human health, and one of the most common forms of malnutrition worldwide is obesity. This pathology, characterized by excessive fat accumulation, is associated with an increased risk of morbidity and mortality (Lettieri Barbato and Aquilano, 2016; World Health Organisation, 2020). In addition, obesity has been linked to meta-inflammation (Rosen and MacDougald, 2006; McGregor and Choi, 2011; Hotamisligil, 2017) which triggers necrosis of adipocytes and releases fatty acids in the blood stream, leading to fat accumulation in other organs, such as the liver (Lumeng and Saltiel, 2011). Lipid accumulation in hepatocytes plays an important role in the onset of various diseases such as non-alcoholic hepatic steatosis (NAFLD), steatohepatitis (NASH), and hepatocellular carcinoma (HCC) (Canbay et al., 2007; Fabbrini et al., 2010; Sun and Karin, 2012; Saitta et al., 2019). Foods contain genetic material that appears to have an epigenetic regulatory activity (Zhang et al., 2012; Baier et al., 2014; Liang et al., 2015; Wagner et al., 2015; Teng et al., 2018; Munir et al., 2020; del Pozo-Acebo et al., 2021). Zhang and collaborators first described the presence of rice miRNAs in sera from humans and animals fed on this cereal (Zhang et al., 2012). In recent years, several studies have suggested that exogenous miRNAs may regulate gene expression between different species (Jiang et al., 2012; Chin et al., 2016; Hou et al., 2018; Zhang et al., 2019). The molecular mechanism underlying this cross-kingdom interaction may explain how diet strongly influences human health and disease development (Cui et al., 2017; Mohammadi et al., 2017; Quintanilha et al., 2017; Li et al., 2021). Several studies suggest that humans ingest exogenous miRNAs through diet and that, once absorbed, they can affect gene expression (Jiang et al., 2012; Zhang et al., 2012; Baier et al., 2014; Liang et al., 2014, 2015; Philip et al., 2015; Title et al., 2015; Zempleni et al., 2015; Zhou et al., 2015; Benmoussa et al., 2016; Quintanilha et al., 2017). There are important examples in the literature of such cross-kingdom interactions. For example, plant miR159a reduced breast cancer growth by inhibiting TCF7 expression in a tumor xenograft model (Chin et al., 2016), and promoted apoptosis in Caco-2 cells (Liu et al., 2020). Two conserved plant miRNAs, miR156, and miR167e-5p, appear to regulate Wnt/β-catenin signaling and maintain homeostasis of the intestinal epithelium, preventing colitis in mice (Li et al., 2019b; 2019a). In addition, strawberry miR168 inhibited T cell proliferation and reduced cytokine release by binding to TLR3 in dendritic cells (Cavalieri et al., 2016). Recently, our group has shown that miRNAs present in dried nuts have an anti-inflammatory effect in mammals by targeting Tumor Necrosis Factor Receptor 1 (TNFR1) in adipose tissue (Aquilano et al., 2019). Moreover, we showed that the introduction of synthetic and natural miRNAs from the drupe *Olea europaea*, restored the function of *hsa*-miR34a, which was deficient in tumor cells (Minutolo et al., 2018). Similarly, natural and synthetic miRNAs from MO were able to modulate the immune system response and to reduce HIV replication (Minutolo et al., 2021). Mankind has always used plants and herbs to treat various diseases. Traditional medicine is still a common remedy in many countries (Wachtel-Galor and Benzie, 2011; Kasilo and Trapsida, 2013), although extreme caution should be exercised as herbal preparations can be harmful to humans in some cases due to the presence of toxic molecules (Shaw et al., 2012; Syed et al., 2015; Mensah et al., 2019). MO is a common plant grown as a food source and has been used in African traditional medicine for centuries (Vergara-Jimenez et al., 2017; Bancessi et al., 2020). MO is considered a famine food because of its high nutrient content and resistance to drought, and every part of the tree is used (Saini et al., 2016; Bao et al., 2020; Mabrouki et al., 2020; Jain et al., 2021). There is increasing interest in the role of non-coding miRNAs in post-transcriptional regulation (Chin et al., 2016; Cui et al., 2017; Lundstrom, 2017; Morales et al., 2017; Hou et al., 2018; Yao et al., 2019). Attention is focused on the cross-kingdom concept which suggests that plant miRNAs may play a key role in regulating the expression of human proteins involved in important cellular processes. Cross-kingdom regulation seems to bring new importance to herbal medicine, as plant miRNAs have shown the ability to regulate perturbed signaling pathways in various pathologies (Sala-Cirtog et al., 2015). With this pilot study, we aim to demonstrate the effect of *mol-*miRs in combating dyslipidemia.

## 2 Materials and methods

### 2.1 *Moringa oleifera* preparations

#### 2.1.1 MO seed aqueous extract preparation

MO mature seeds were harvested in the Dschang District, West Cameroon (Africa) by the Cooperative of Medical Plant Producers SOCOPOMO. The seeds were sun-dried and stored until use. In our laboratory, the seeds were hulled and ground in a mortar to a fine powder, according to the traditional preparation. MO powder was then boiled in distilled water for 15 min. After cooling, the mixture was subjected to several centrifugations to remove solid residue. The MOES was then syringe filtered (0.45 µm, Minisart®) and stored at −20°C until use (Potestà et al., 2019).

#### 2.1.2 MO miRNAs extraction

MO miRNAs (*mol*-miRs) were extracted from MOES using the NucleoSpin® miRNA kit (MACHEREY-NAGEL, Germany). *mol*-miRs were quantified using a NanoDrop^tm^ Light Spectrophotometer (Thermo Fisher Scientific, United States). The presence of the main miRNAs (miR-156a, miR-159a, miR-159c, miR-160h, miR-162a, miR-166i, miR-167-5p, miR-171a, miR-393, miR-395a, miR-396c, miR-397, miR-398, miR-482b, miR-858a, miR-858b, miR-2118a) in the *mol*-miR pool was confirmed by quantitative RT-PCR (qRT-PCR) as previously reported (Gismondi et al., 2017; Minutolo et al., 2018; Potestà et al., 2019, 2020).

### 2.2 Cell culture

The human HepG2 hepatoma cell line was grown in Dulbecco’s modified Eagle’s medium (DMEM) supplemented with 10% foetal bovine serum, 100 U/ml penicillin, 100 mg/ml streptomycin and 2 mM L-glutamine (Lonza, United States). For maintenance, cells were harvested with a mixture of 170,000 U Trypsin/L and 200 mg/L Versene (EDTA) (Lonza, BioWhittaker™) and reseeded in fresh medium as a function of cell density.

### 2.3 MOES treatment

0.5 × 10^5^ HepG2 cells/mL were treated for 72 h with MOES at different concentrations (2.5 mg/ml, 5 mg/ml and 10 mg/ml). Cells were washed with PBS, collected, centrifuged and the pellets stored at −20°C for subsequent analyses.

### 2.4 *mol*-miR pool transfection

HepG2 cells were transfected with the *mol*-miR pool at a concentration of 5 μg/ml using lipofectamine (Hi-Fect, Qiagen, HF). Control HepG2 cells were treated with lipofectamine alone.

### 2.5 Cell viability and apoptosis

Cell viability was assessed using the Trypan Blue (Euroclone S.P.A. ITA) exclusion test. Viable and dead cells were counted using the Neubauer chamber. Apoptosis was assessed by flow cytometry analysis of isolated nuclei stained with Propidium Iodide (Sigma) using a CytoFLEX flow cytometer (Beckman Coulter, United States). Data acquisition and analysis were performed using CytExpert 2.2 (Beckman Coulter, United States). At least 150,000 events were counted for each sample.

### 2.6 Lipids and TNF-alpha intracellular staining

BODIPY (BPI) (Thermo Scientific) was used to evaluate the accumulation of lipid droplets in HepG2 cells, as previously described (Minutolo et al., 2018). The analysis was performed using a CytoFLEX flow cytometer. The gating strategy is shown in Supplementary Figure S2. TNF-alpha expression was evaluated by flow cytometry analysis. Transfected HepG2 cells stained with FITC Mouse Anti-Human TNF (BD Biosciences, Franklin Lakes, NJ, United States) were analyzed using a CytoFLEX flow cytometer (Beckman Coulter, United States) and CytExpert 2.2 software. 10,000 events were counted in live cells gate.

### 2.7 Computational prediction of miRNA-mRNA interactions

The prediction tool used in this work is a support vector machine (SVM)-based classifier, trained on an experimentally validated set of miRNA-mRNA interactions. This classifier discriminates potential miRNA-mRNA interactions by combining the output of nine different RNA-RNA prediction algorithms (Minutolo et al., 2021). We developed a code to automatically run the programs and generate an energy-based probability score (ES) and a probability-based score (PS) as output. The final ES score was obtained by reformulating the Fermi-Dirac Eq. (1):

$$ES=\overset{N}{\underset{i}{\sum }}\frac{1}{1+e^{\left(E_{i}\right)/RT}}$$ (1)

where E_i_ represents the interaction energy of the miRNA-mRNA pair and RT is the universal gas constant. The probability scores are simply summed using Eq. (2).

$$PS=\overset{N}{\underset{i}{\sum }}{SCORE}_{i}$$ (2)

The prediction codes and scoring functions were applied to a training set and a test set consisting of experimentally validated miRNA-mRNA interactions and used to build a classifier through the *sklearn. svm. SVC* function of the Python *scikit-learn* package (Pedregosa et al., 2011). The full description of the SVM classifier implementation is reported in our previous work (Minutolo et al., 2021).

### 2.8 *In vivo* mice experiments

*In vivo* experiments performed in accordance with accepted standards for the care and use of laboratory animals after the approval by the University Ethics Committee for Animal Experimentation (Institutional Animal Care and Use Committee—IACUC, Tor Vergata University) and national committees (Ministry of Health) with authorization n°378/2017-PR. A total of 12 young, 2 month-old male C57BL/6J mice (purchased from Harlan Laboratories S.r.l., 91 Urbino, Italy) were randomly divided into two groups (6 mice/group): mice fed a normal calorie diet (ND) (3.85 kcal/g among which 10% kcal from fat, 20% from protein and 70% from carbohydrate) and mice fed a high fat diet (HFD: 5.24 kcal/g among which 60% kcal from fat, 20% from protein, and 20% from carbohydrate). Each group (ND + *mol-*miR and HFD + *mol-*miR) was administered 3 µg of the *mol-*miR pool resuspended in RNA-free water orally by gavage every 2 days. The dose required for the *in vivo* study was chosen considering previous toxicological *in vitro* studies performed in murine cell lines (data not shown) and the average mouse blood volume (about 3 ml): 3 µg/mice/3 times per week *mol*-miR pool. The total dietary treatment was maintained for 5 weeks. The body weight of mice was measured every week from the start of the treatment (T_0_), and the weight change was assessed as delta between two consecutive time points (T_X_-T_0_). At the end of the treatments (5 weeks), mice have been sacrificed by cervical dislocation, organs harvested, weighed, and tissues were prepared for subsequent analysis.

### 2.9 Biochemical parameters

For serum protein electrophoresis, blood samples were collected in SST microtainers (Serum Separator Tube; Dickinson Company, Boston, United States) and centrifuged at 13,000 g for 7 min. For the measurement of blood glucose, mice were fasted overnight before blood sampling. Glucose and total cholesterol were measured using the automatic analyzer Keylab (BPC BioSed S.r.l., Rome, Italy) according to the manufacturer’s instructions.

### 2.10 Histological analysis

For histological analysis mouse livers were fixed in 10X formalin and embedded in paraffin. Hematoxylin and Eosin (H&E) staining was performed to evaluate the inflammatory state of liver tissues.

### 2.11 RNA extraction

Total RNA was extracted from mouse livers and HepG2 cells using TRIzol extraction, according to the manufacturer’s instructions (Invitrogen, CA). RNA was quantitated using a NanoDrop^tm^ Light Spectrophotometer (Thermo Fisher Scientific, United States) and stored at −20°C until use.

### 2.12 Reverse transcription

cDNA synthesis was performed using a high-capacity cDNA Reverse Transcription Kit (Applied Biosystem by Life Technologies NY, United States Invitrogen, CA) according to the manufacturer’s instructions. The cDNA was stored at 4°C until use.

### 2.13 Adipogenesis RT^2^ profiler PCR array

The expression of 84 metabolic genes involved lipid metabolism was analyzed in HepG2 cells treated with MOES 2.5 mg/ml, and mouse liver tissues exposed to *mol*-miR pool for 5 weeks. Analysis was performed on the cDNA obtained from total RNA using Human Adipogenesis RT^2^ Profiler PCR Array (PAHS-049Z; Qiagen) and Mouse Adipogenesis RT^2^ Profiler PCR Array (PAMM-049Z; Qiagen), respectively. A 7500 Fast Real-Time kit (Applied Biosystems) with SYBR Green detection (Hydra SYBR qPCR Master Mix, BIOLAB) was used according to the manufacturer’s instructions. Data elaboration was performed using the RT^2^ Profiler PCR Array Data Analysis software. Transcriptional levels were shown as fold change (HFD vs. ND; HFD + *mol*-miR pool vs. ND; HFD group vs*.* HFD + *mol*-miR pool). Values > 1.50 units or <0.5 units were considered significant. All dataset was submitted to Geo Repository http://www.ncbi.nlm.nih.gov/geo. Accession number: GSE125344 (human), GSE125346 (mouse).

### 2.14 MO miRNAs purification

*mol*-miRs were purified from mice liver total RNA using the NucleoSpin® miRNA kit (MACHEREY-NAGEL, Germany). *mol*-miRs were quantified using a NanoDrop^tm^ Light Spectrophotometer (Thermo Fisher Scientific, United States). The *mol*-miR pool was characterized by quantitative RT-PCR (qRT-PCR). The presence and concentration of the most conserved miRNAs were validated and measured as previously reported (Gismondi et al., 2017; Minutolo et al., 2018; Potestà et al., 2019, 2020). A Bio-Rad thermal cycler (IQ5) was used and amplification parameters were set according to the instructions of EXIQON pre-designed primers, as previously reported (Potestà et al., 2019, 2020). Relative expression of miRNAs was quantified using the 2^−ΔΔCt^ method, using 5S RNA as internal control.

### 2.15 Statistical analysis

Data are presented as mean ± standard deviation (SD). All experiments were repeated at least in triplicate (biologically independent measurements). To compare the means of two different groups, the unpaired Student’s t-test was used. For comparison of means of more than two groups, the nonparametric one-way ANOVA with the Kruskal-Wallis test was used. Pre-hoc statical analysis was performed to determine the sample size, we considered a power analysis of 0.8 and assumed an effect size of 4.0, based on the 30% difference in blood glucose levels between mice fed with ND and HFD (Aquilano et al., 2019). A sample size of *n* = 3 mice in each group (α error = 0.05 and β = 0.2) was estimated to reject the null hypothesis of no difference (http://www.statisticalsolutions.net/pss_calc.php). A nonparametric one-way post-hoc ANOVA corrected by Kruskal-Wallis test, was used for the statistical analysis of animal experiments. *p*-value < 0.05 (*), *p* < 0.01 (**) or *p* < 0.001 (***) were considered significant.

## 3 Results

### 3.1 Effect of MOES in HepG2 cells

**FIGURE 1:**
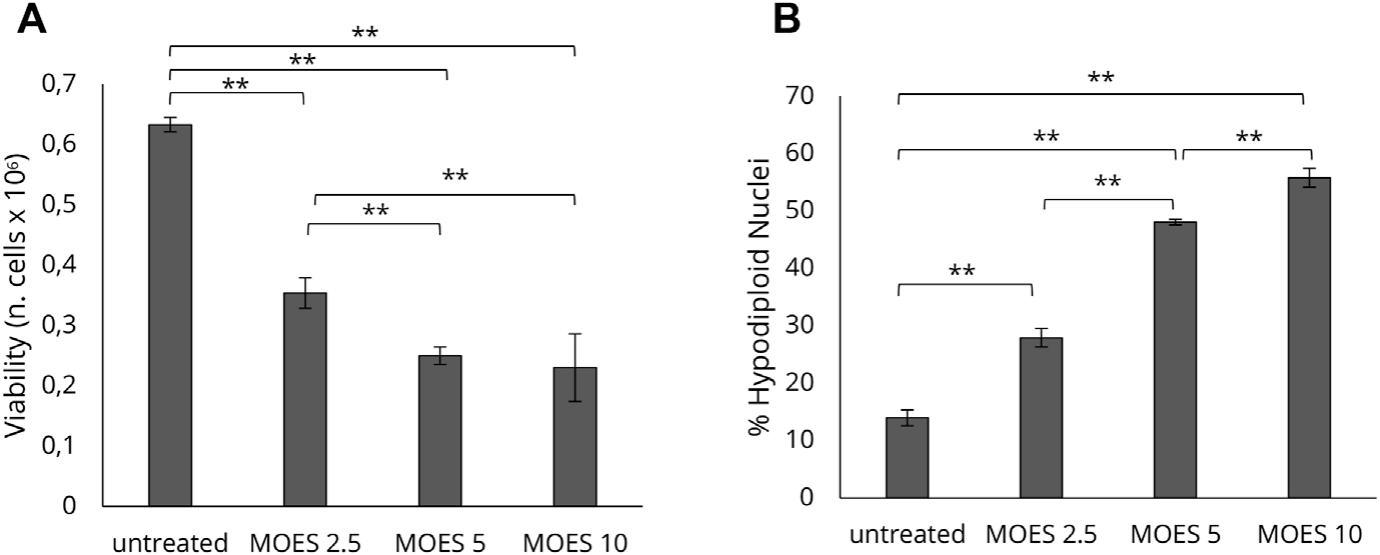
Dose-response effect of MOES on the HepG2 cell line. The effect at increasing concentrations (0, 2.5, 5, 10 mg/ml) of MOES on HepG2 cell viability and apoptosis. **(A)** The number of viable cells analysed by Trypan Blue assay after 72 h of treatment. **(B)** Percentage of apoptotic cells after 72 h of treatment, evaluated by propidium iodide staining and flow cytometry analysis. All results derived from, at least, three independent biological experiments (sample size *n* = 9). Histograms report the mean ± SD (**p < 0.01). Unpaired Student’s t-test was used.

**FIGURE 2:**
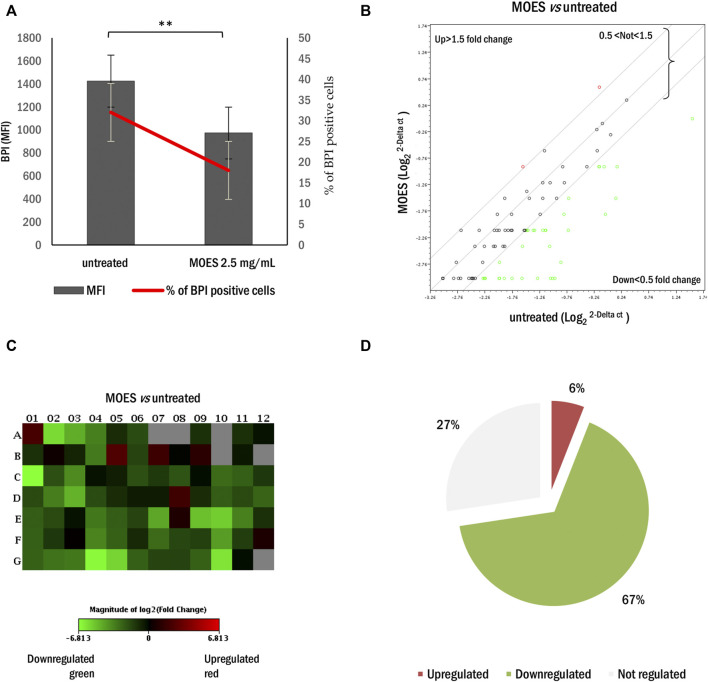
Effect of MOES on lipid metabolism in the HepG2 cells. **(A)** MFI (black column) and percentage (red line) of BPI positive HepG2 cells treated with MOES. All results derived from at least three independent biological experiments. The histogram reports the mean ± SD (**p < 0.01). Unpaired Student’s t-test was used. **(B)** The scatter plot array profile of genes involved in lipid metabolism in HepG2 cells. Elaboration by RT^2^ Profiler PCR Array Data Analysis software. Values > 1.50 or <0.5 were considered significantly upregulated (red dots) and downregulated (green dots), respectively. **(C)** The relative Heat Map shows the expression of upregulated (red) and downregulated (green) genes in HepG2 treated with MOES. **(D)** The number of genes and their percentage values, regulated by MOES, represented as Pie Chart.

To investigate the effects of MOES on the hepatocellular carcinoma HepG2 cell line, we first determined an appropriate dosage for treatment. Cells were treated with three concentrations of MOES: 2.5, 5, and 10 mg/ml for 72 h, as previously reported in a toxicology study (Potestà et al., 2019). After 72 h, MOES significantly decreases the number of viable cells (Figure 1A) and increased the number of apoptotic cells as evidenced by a dose-dependent increase of hypodiploid nuclei percentage (Figure 1B). At a MOES concentration of 2.5 mg/ml, a significant decrease in cell viability (about 20%) and an increase in hypodiploid nuclei were observed compared with controls (Table 1). At higher concentrations, treatment leads to a progressive decrease in cell number. Based on these results and previous results obtained with other cell lines (Potestà et al., 2019, 2020), 2.5 mg/ml MOES was used for further investigations. To determine whether MOES affects hepatic lipid accumulation, a typical feature of HCC and hepatic steatosis (Liu et al., 2010; Gu et al., 2016), HepG2 cells were stained with BPI and analyzed by flow cytometry. A significant decrease in Mean Fluorescence Intensity (MFI) and percentage of BPI positive cells was observed in treated cells compared with controls (Figure 2A and Table 1), indicating an ability of MOES to reduce intracellular lipid accumulation. Using a Human Adipogenesis RT^2^ Profiler PCR Array we found that MOES can modulate the expression of 84 genes linked to lipid metabolism. Comparing control and treated cells, 6% of the genes examined were upregulated, whereas 67% were downregulated (Figures 2B–D). Among the downregulated genes we found members of various metabolic pathways involved in tumorigenic and inflammatory processes (Supplementary Table S1).

**TABLE 1:** Effect of MOES on HepG2 cells.

*In vitro* model				CTR	MOES 2.5	MOES 5	MOES 10
HepG2	Viability	Trypan Blue assay	Fold Change Treated vs*.* Untreated	0.63 ± 0.01	0.35 ± 0.02	0.25 ± 0.015	0.23 ± 0.032
	Apoptosis	Propidium Iodide	% Hypodiploid nuclei	13.95 ± 2.31	27.89 ± 3.54	48.04 ± 5.45	55.75 ± 6.56

Mean ± SD of three independent measurement.

### 3.2 Computational prediction of *mol*-miRs putative genes target in human

Based on our previous work, the modulatory activity of MOES was associated with the presence of *mol*-miRs in this extract (Potestà et al., 2020; Minutolo et al., 2021). Therefore, a bioinformatic analysis has been carried out searching for potential interactions between *mol*-miRs and genes involved in lipid metabolism. The prediction pipeline was based on an SVM classifier, trained on an experimentally validated set of miRNA-mRNA interactions, combining the output of several miRNA-mRNA interaction prediction algorithms implemented in our previous work (Minutolo et al., 2021)*.* For the analyses, we selected the same gene panel that was used for the Adipogenesis RT^2^ Profiler PCR Array. Interestingly, the prediction pipeline identified several *mol*-miRs in the MOES with a high probability of interacingt with the genes modulated by treatment in HepG2. As shown in Supplementary Table S1, this analysis indicated that of the 61 genes modulated by MOES, 22 were potential targets of *mol*-miRs in humans (with a greater than 60% probability of being assigned to the correct class).

### 3.3 Effects of *mol*-miR pool on viability, apoptosis, and lipid accumulation in HepG2 cells

**FIGURE 3:**
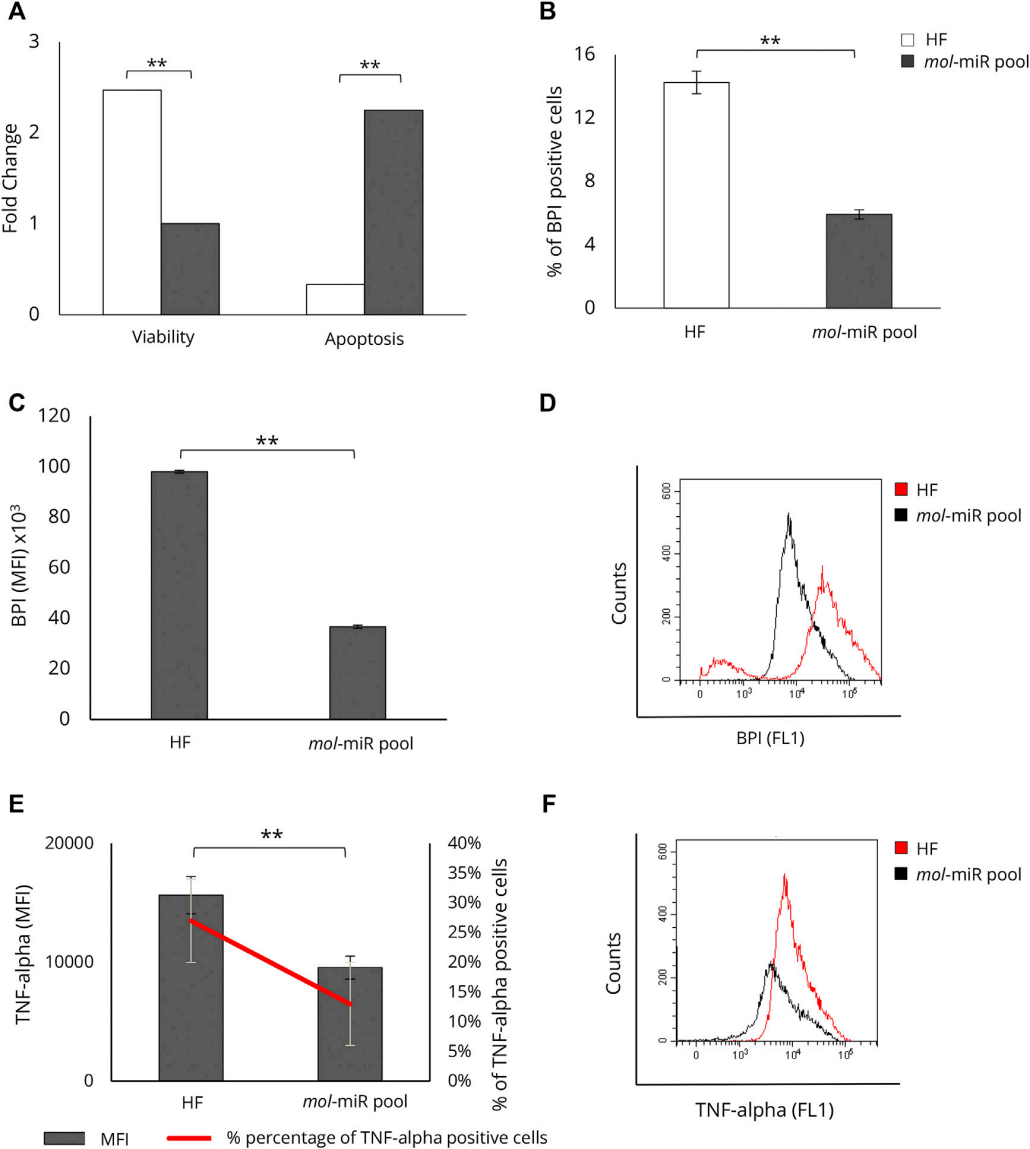
Characterization of *mol*-miR pool effect on HepG2 viability, apoptosis, and lipid accumulation. **(A)** Effect of *mol*-miR pool transfection on apoptosis and viability in HepG2 cells. **(B)** Percentage of BPI positive HepG2 cells treated with *mol*-miR pool. **(C)** MFI of BPI positive HepG2 cells treated with *mol*-miR pool. **(D)** Representative overlay histogram of lipid content in HepG2 cells treated with *mol*-miR pool. **(E)** MFI (black column) and percentage (red line) of TNF-alpha positive cells. **(F)** Representative overlay histogram of TNF-alpha in HepG2 cells treated with *mol*-miR pool. All results derived from at least three independent biological experiments (sample size *n* = 9). Histograms report the mean ± SD (**p < 0.01). Unpaired Student’s t-test was used.

We tested whether the *mol-*miR pool present in MOES could be the mediator of the biological effects observed in HepG2 cells. The effects of the *mol-*miR pool on cell viability and apoptosis were evaluated and compared with those induced by MOES. The *mol-*miR pool significantly reduced cell viability and increased apoptosis in HepG2 cells (Figure 3A). In addition, *mol-*miRs cause a significant decrease in both the percentage of BPI positive cells (Figure 3B) and MFI together with a decrease in intracellular lipid levels, compared to controls (Figures 3C,D). These results suggest that the *mol-*miR pool, like the MOES, is able to reduce cell proliferation and increase apoptosis by interfering with accumulation of lipids in HepG2 cells (Table 2). These processes are associated with inflammation, and TNF-alpha plays a key role as a pleiotropic pro-inflammatory cytokine (Jing et al., 2018; Sethi and Hotamisligil, 2021) We have recently shown that two conserved plant miRNAs, miR159a and miR156c, can impair TNF-alpha signaling by reducing inflammation (Aquilano et al., 2019). This finding was also confirmed in the present model, as we found that treatment with *mol-*miR pool, containing miR159a and miR156c, significantly reduces MFI and the percentage of TNF-alpha positive HepG2 cells (Figures 3E,F and Table 2).

**TABLE 2:** Effect of *mol-*miRs on HepG2 cells.

*In vitro* model				CTR	*mol*-miRs
HepG2	Viability	Trypan Blue assay	Fold Change Treated vs*.* Untreated	2.46 ± 0.11	0.35 ± 0.01
	Apoptosis	Propidium Iodide	Fold Change Treated vs*.* Untreated	1	2.25 ± 0.08
	Lipid Accumulation	BODIPY^TM^	Mean intensity of fluorescence	1,424 ± 965	592 ± 58
			% Positive cells	32 ± 9	14.24 ± 3.4
	Inflammation	TNF-alpha	Mean intensity of fluorescence	15,658 ± 932	9,568 ± 641
			% Positive cells	27 ± 9	13 ± 5

Mean ± SD of three independent measurement.

### 3.4 Computational prediction of *mol*-miRs putative target genes in mice

Before examining the effects of *mol*-miRs in the mouse model, we applied the prediction pipeline to search for their putative mRNA targets in *Mus musculus*. The analysis considered the same set of genes used for the analyses in *Homo sapiens*. The SVM classifier identified 41 genes as potential targets of *mol-*miRs (with a greater than 60% probability of being assigned to the correct class), 19 of which were consistent with *Homo sapiens,* as shown in Table 3. Of these 19 target genes, 17 were downregulated and 2 were upregulated by MOES treatment containing *mol*-miRs (Supplementary Table S2).

**TABLE 3:** List of the nineteen *mol-*miRs target genes in common between *Homo sapiens* and *Mus musculus,* with relative *mol-*miRs and binding energy shown as score.

Genes	Description	Pathway	*mol*-miRs predicted for human genes	*mol*-miRs predicted for murine genes
AGT	Angiotensinogen	Hormones	miR160h (0.65), miR395d (0.96), miR482b (0.97)	miR160h (0.90), miR159c (0.90), miR167f-3p (0.96), miR397a (0.68)
CDKN1A	Cyclin-dependent kinase inhibitor 1A	Tumorigenesis	miR395d (0.93), miR160h (0.96), miR166 (0.97)	miR160h (0.96)
CFD	Complement factor D	Adipokines	miR160h (0.95)	miR160h (0.97)
DIO2	Deiodinase, iodothyronine, type II	Pro-Brown Adipose Tissue	miR166 (0.96), miR396a (0.99)	miR159c (0.94), miR156e (0.94), miR166 (0.87)
EGR2	Early growth response 2	Pro-White Adipose Tissue	miR166 (0.60), miR159c (0.82)	miR167f-3p (0.95), miR159c (0.94)
INSR	Insulin receptor	Pro-Brown Adipose Tissue	miR396a (0.85), miR482b (0.98)	miR395d (0.90)
KLF2	Kruppel-like factor 2	Anti-White Adipose Tissue	miR2118a (0.87), miR482b (0.96), miR166 (0.97)	miR166 (0.97), miR160h (0.88)
LEP	Leptin	Adipokines	miR159c (0.95), miR482b (0.96), miR160h (0.96), miR166 (0.98)	miR395d (0.95), miR159c (0.91), miR160h (0.92)
LPL	Lipoprotein lipase	Enzymes	miR160h (0.61)	miR167f-3p (0.65)
MAPK14	Mitogen-activated protein kinase 14	Pro-Brown Adipose Tissue	miR393a (0.92), miR396a (0.98)	miR159c (0.93)
NRF1	Nuclear respiratory factor 1	Pro-Brown Adipose Tissue	miR160h (0.94), miR396a (0.97), miR482b (0.98)	miR167f-3p (0.85)
PPARA	Peroxisome proliferator receptor alpha	Beta-Oxidation	miR393a (0.75), miR160h (0.96), miR166 (0.96)	miR397a (0.96), miR482b (0.94), miR160h (0.87)
RB1	Retinoblastoma 1	Anti-Brown Adipose Tissue	miR160h (0.86)	miR159c (0.61)
RXRA	Retinoid X receptor alpha	Cholesterol Metabolism & Transport	miR160h (0.97), miR166 (0.98)	miR160h (0.93), miR159c (0.93), miR398a-5p (061), miR166 (0.61)
SFRP1	Secreted frizzled-related protein 1	Pro-Adipogenesis	miR160h (0.84), miR166 (0.84), miR482b (0.97)	miR160h (0.92), miR166 (0.92)
SHH	Sonic hedgehog	Tumorigenesis	miR159c (0.95), miR482b (0.98)	miR858b (0.96), miR167f-3p (0.72)
VDR	Vitamin D receptor	Tumorigenesis	miR160h (0.71), miR166 (0.97)	miR160h (0.91), miR398a-5p (0.91), miR166 (0.63)
WNT3A	Wingless-related MMTV integration site 3A	Tumorigenesis	miR171b (0.87), miR166 (0.92), miR482b (0.94), miR2118a (0.96), miR160h (0.98)	miR159c (0.93), miR395d (0.85)
WNT10B	Wingless related MMTV integration site 10b	Tumorigenesis	miR166 (0.62), miR167f-3p (0.88), miR395d (0.96), miR482b (0.96), miR2118a (0.96), miR160h (0.98)	miR858b (0.96), miR167f-3p (0.94), miR160h (0.91)

### 3.5 Effect of *mol*-miR pool on mechanisms associated with lipid accumulation in a pre-obese mice model

**FIGURE 4:**
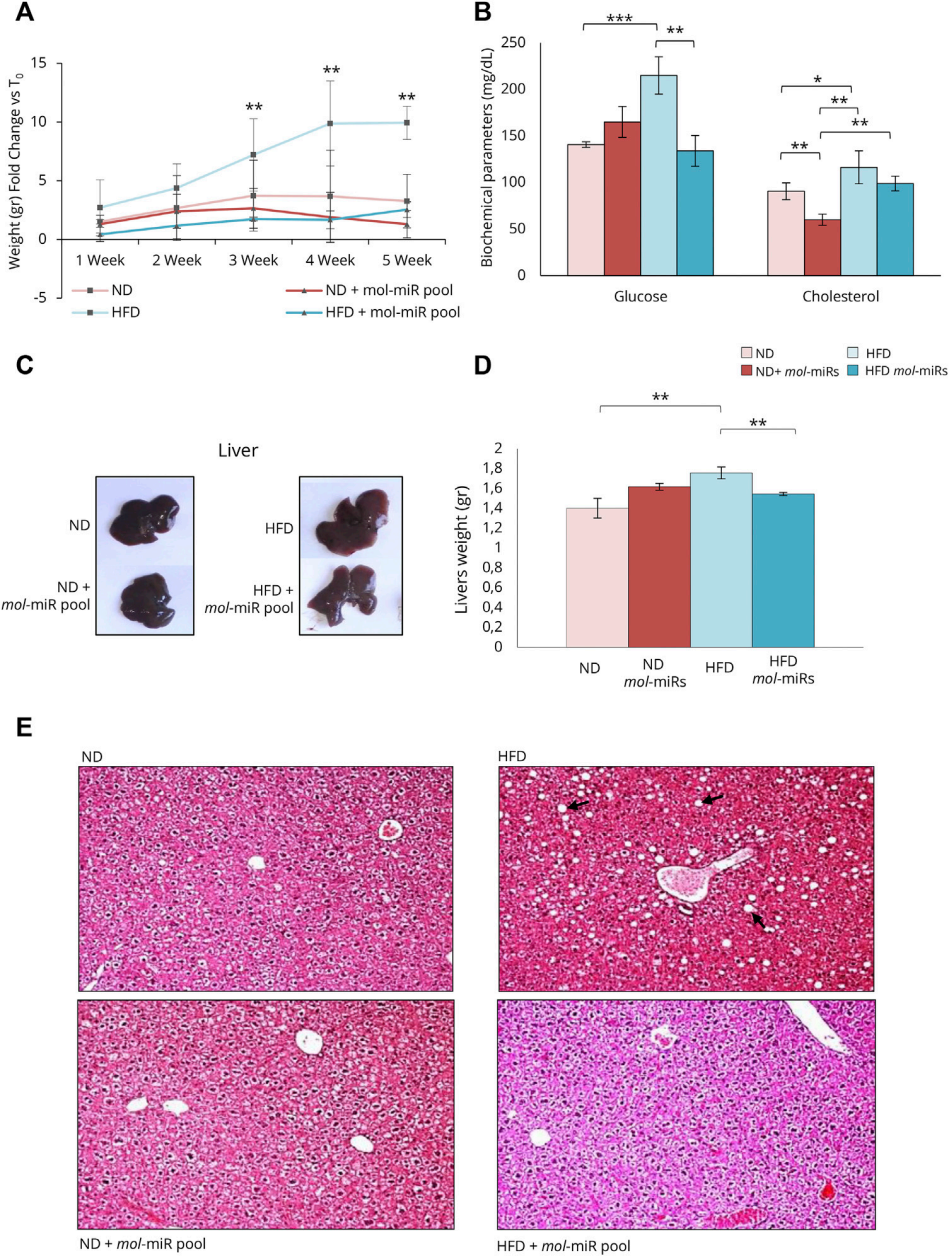
Effect of *mol*-miR pool in mice after High Fat Diet (HFD). **(A)** Delta of mouse body weight (grams). The mouse body weight was measured every week from the start of the treatment (T_0_). **(B)** Biochemical parameters in mice blood measured after 5 weeks. **(C)** An overview picture of mice livers. **(D)** Livers weights (grams). Histograms report the mean ± SD (*n* = 3 each group, **p < 0.01). Nonparametric one-way ANOVA corrected by the Kruskal-Wallis test was used. **(E)** Pictures of H&E staining of liver sections of ND or HFD mice supplemented and not with *mol*-miR pool. The black arrows in the HFD panel, indicate the lipid droplets accumulated in the hepatocytes.

Hence, an *in vivo* study on C57BL/6J mice was conducted to elucidate the possible anti-inflammatory effect of the *mol*-miR pool and its ability to modulate lipid accumulation. Mice were fed HFD for 5 weeks to induce a pre-obese state and the effects of *mol-*miRs pool treatment on lipid metabolism were evaluated. HFD feeding led to an increase in body weight compared with mice fed ND. The HFD + *mol-*miR pool group exhibited similar body weight gain as the ND group (Figure 4A and Table 4). Furthermore, the HFD + *mol-*miR pool group had lower glucose levels than the HFD group and this level was comparable to that of the ND group. Although no significant difference in cholesterol levels was found between the HFD + *mol*-miRs and HFD groups, of the plasma cholesterol concentration in the HFD + *mol*-miRs group decreased compared with that in HFD mice; moreover, the cholesterol level in the HFD + *mol*-miRs group was similar to that in the ND group (Figure 4B and Table 4). In pre-obese and obese subjects, lipids gradually accumulate in various organs, with the liver being the first organ affected by excessive lipid accumulation (Ahmed et al., 2014; De Mello et al., 2018). As shown in Figures 4C,D and reported in Table 4, the weight of the livers of HFD mice were significantly higher than that of the livers of the HFD + *mol-*miR pool group (**p < 0.01). In addition, H&E staining of liver sections revealed marked lipid accumulation in HFD mice (Figure 4E), reflecting the lipid accumulation typical of overweight conditions. Lipid accumulation was not observed in HFD + *mol-*miR pool and in control groups.

**TABLE 4:** Effect of *mol-*miRs on mice.

*In vivo* model			ND	ND + *mol*-miRs	HFD	HFD + *mol*-miRs	Range value
C57BL/6J	Body weight	gr	3,26 ± 4.27	1,30 ± 1.17	11,6 ± 4.41	2,53 ± 0.66	
	Liver weight	gr	1.4 ± 0.59	1.52 ± 0.32	1.75 ± 0.07	1.54 ± 0.07	
	Glucose	mg/dL	140.66 ± 3.05	165 ± 36	215 ± 19,97	134 ± 16	60–130
	Cholesterol	mg/dL	90.66 ± 9.07	36 ± 4.24	116.33±	99 ± 18.92	50–120

Mean ± SD of three independent measurement.

### 3.6 Lipid metabolism gene profile of mice livers

**FIGURE 5:**
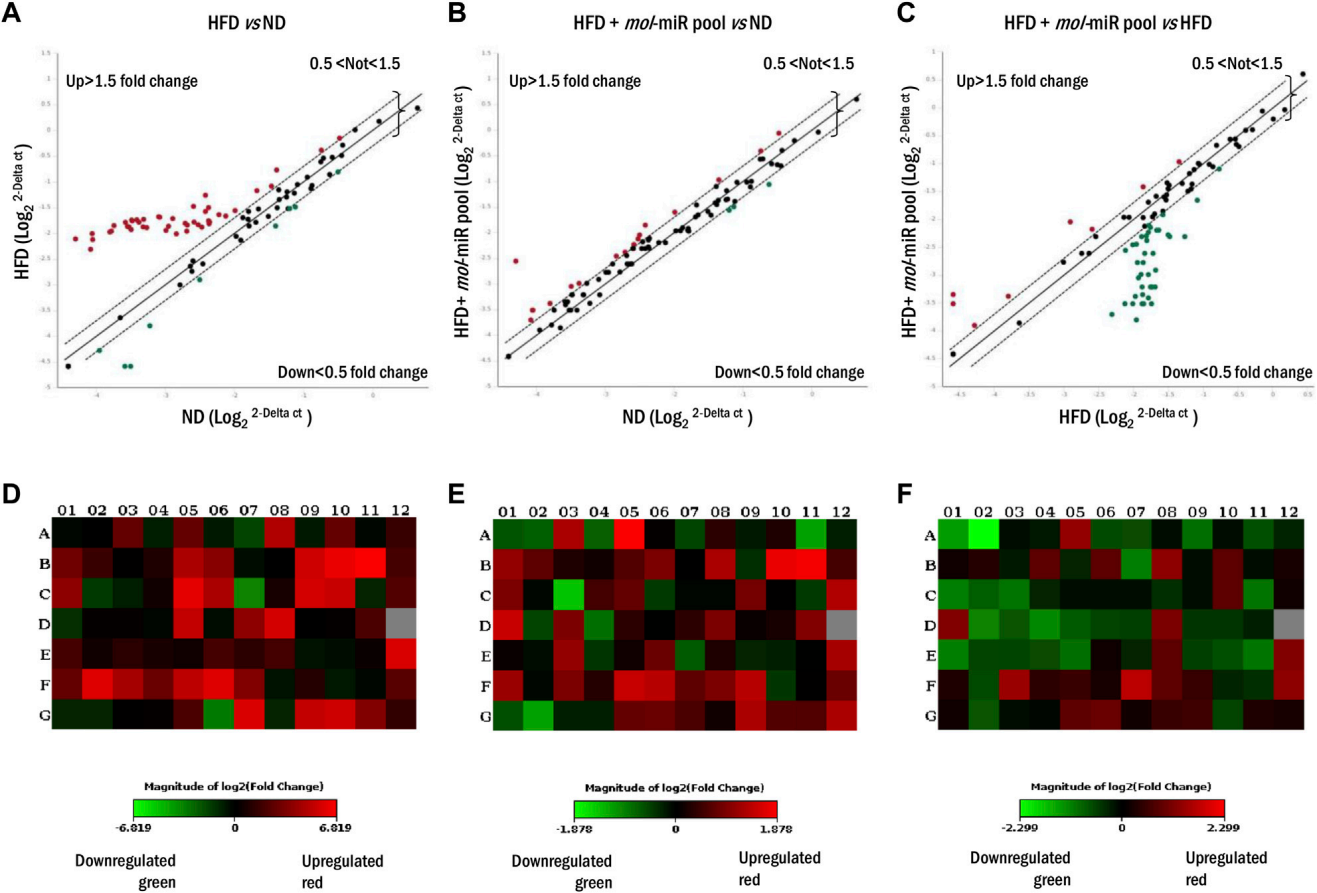
Lipid metabolism gene expression profile in livers of mice fed for 5 weeks with HFD and HFD + *mol*-miR pool. **(A–C)** Liver scatter plot array profile. Transcriptional levels of specific genes are shown as fold change. Values > 1.50 or <0.5 were considered significantly upregulated (red dots) and downregulated (green dots), respectively. Values between 1.5 and 0.5 were considered not regulated. **(D–F)** The relative Heat Map shows the expression of upregulated (red) and downregulated (green) genes in livers of mice subjected to **(D)** HFD vs*.* ND group, **(E)** HFD + *mol*-miR pool vs*.* ND group, **(F)** and HDF + *mol*-miR pool vs*.* HFD group.

Because prolonged HFD leads to alterations in liver morphology and function, the expression of genes related to lipid metabolism was examined using microarray RT-PCR analysis (Figure 5). The livers of HFD mouse showed an upregulation of genes involved in the control of lipid metabolism compared to those of ND mice (Figure 5A). Of the 84 genes comprising the array, 45 (53.6%) were significantly upregulated in HFD mice and only 7 (8.3%) were downregulated (Figures 5A,D). The HFD + *mol*-miRs group showed an altered pattern compared with the ND group with 16 (19.1%) genes were significantly upregulated, and 3 (3.6%) genes were downregulated (Figures 5B,E). Finally, the HFD + *mol*-miR pool group showed significant downregulation of gene expression compared with the HFD group. Thirty-nine genes (46.4%) were downregulated (i.e. Leptin, Resistin, Gata2, Gata3, Klf2, Ppra, Pprd, Wnt3a, Wnt5a, Wnt10b) and eight (5.5%) were upregulated (Figures 5C,F and Supplementary Table S3). These data suggest that the *mol-*miRs can reduce HFD-induced lipid dysmetabolism by regulating gene expression.

### 3.7 Detection of *mol-*miRs in mouse livers

To exert their regulatory effect, *mol*-miRs must reach the target tissue after they are supplied with food. To confirm the presence of *mol*-miRs in mouse livers, RT-qPCR analysis was performed. Twenty *mol-*miRs, which are among the most conserved plant miRNA families were analyzed, and seven of them were identified in significant amounts in the livers of both ND and HFD mice treated with *mol*-miRs. Significantly lower amounts of *mol*-miRs (Table 5) were detected in the livers of the HFD + *mol*-miR pool group compared with the ND + *mol*-miR pool group, most likely due to their regulatory activity counteracting the effect of HFD and leading to degradation of the mRNA to which they are bound.

**TABLE 5:** The *mol*-miRs relative level in mouse livers, quantified by qPCR, and normalized with the housekeeping gene 5S rRNA.

	miR159c	miR156a	miR162a	miR171d	miR482b	miR397-5p	miR166i
HFD	0.213*	0.06*	2.044*	0.024***	0.11**	0.03***	00.001***
ND + pool	84.54**	35.91**	50.27**	184.39***	34.65**	145.00***	98.01***
HFD + pool	19.15**^++^	6.75*^++^	10.92**^++^	21.75**^++^	8.18*^++^	32**^++^	36.92**^+^

*p < 0.05, **p < 0.01 and ***p < 0.001 treated vs*.* ND. + *p*< 0.05 and ++*p*< 0.01 HFD + *mol*-miR pool vs*.* ND + *mol*-miR pool.

## 4 Discussion

Functional foods are defined as products rich in molecules that provide health benefits and, when included in a balanced diet, have preventive health benefits such as improving immune response, reducing the risk of disease and comorbidities, and may play an important role in weight control (Hodas et al., 2021). Many functional foods and nutraceuticals are derived from traditional medicine in various countries, and *M. oleifera* Lam. is one example. In this work, a model of lipid dysmetabolism and its treatment with a plant-based remedy based on an African ethnomedicine was evaluated. The lipid modulatory properties of MO are widely recognized (Barbagallo et al., 2016; Almatrafi et al., 2017; Oyeleye et al., 2019; Ezzat et al., 2020). In our studies, we focused on the use of miRNAs present in the MOES (Cui et al., 2017; Mohammadi et al., 2017; Quintanilha et al., 2017; Potestà et al., 2020; Minutolo et al., 2021) and their biological effects. First, the modulatory property of MO was investigated in HepG2 cells, an *in vitro* model of liver tissue. Treatment with *mol-*miRs was able to decrease lipid accumulation and induce apoptosis in this cell line. Furthermore, bioinformatic analysis assessed the ability of *mol*-miRs to interact with human and mouse genes involved in lipid metabolism and tumorigenesis. It is important to note that plant derived miRs share functional homology rather than sequence homology with mammalian miRs (Minutolo et al., 2018). The analysis evaluated the probability of interaction and binding energy between the *mol*-miRs and the mRNA of the genes analyzed. Interestingly, bioinformatic analysis confirmed the ability of *mol*-miRs to modulate gene expression in both humans and mice. In view of these results, a pilot study was performed in C57BL/6J mice. A pre-obese state was induced in the mice by HFD feeding, and interestingly, we found that supplementation with the *mol*-miR pool reduced weight gain. Consistent with this finding, genes that were upregulated by HFD diet were downregulated by *mol*-miR pool supplementation, suggesting a modulatory effect of plant miRNAs. The liver plays key role in the regulation of lipid homeostasis, and obesity is strongly associated with liver abnormalities (Fabbrini et al., 2010; Sun and Karin, 2012; Saitta et al., 2019). HFD mice exhibit lipid accumulation in the liver that was not observed in HFD + *mol*-miR pool mice. The miRNAs present in the *mol*-miR pool were also detected in the liver tissue of mice not treated with *mol-*miRs (Table 5). This apparent inconsistency is due to the diet of the mice that is predominantly plant-based and therefore contains such miRNAs. Importantly, supplementation with *mol-*miRs significantly increased the presence of plant miRNAs in mouse livers, confirming that they entered this tissue. There was a significant increase in these miRNAs in ND + *mol*-miR pool mouse livers, although to a lesser extent that in HFD + *mol*-miR pool livers. Presumably the *mol-*miRs bound to the mRNAs and exerted their regulatory effect. The lowest amount of *mol*-miRs detected in the liver of HFD mice (Table 5) also suggests that the higher degradation of *mol*-miRs may be due to a longer delay in gastric emptying because of the high-fat content of the diet (Cunningham et al., 1991; Lettieri Barbato et al., 2014). The expression of important genes involved in both lipid metabolism and tumorigenesis was studied in mouse livers. Array analysis showed that genes generally upregulated in obesity were also upregulated in HFD mouse livers. Interestingly the HFD + *mol*-miR pool group shows a gene expression pattern analogous to the ND group (Supplementary Table S3 and Figure 5), demonstrating the regulatory activity of the *mol-*miR pool. Adipose tissue is a complex endocrine organ that contributes to the regulation of whole-body homeostasis (Gregor and Hotamisligil, 2011; Coelho et al., 2013; Leal and Mafra, 2013). The tissue actively secretes adipokines that act locally and systemically (Kershaw and Flier, 2004; Booth et al., 2016). Excessive adiposity leads to dysregulation of these cytokines, resulting in metabolic inflammation (Lettieri Barbato et al., 2014). The inflammation triggered by obesity is a peculiarity that manifests itself in a low-grade activation of innate immune responses that, over time, affect steady-state measures of metabolic homeostasis, and have a pro-inflammatory effect in multiple organs (Hotamisligil, 2006; Lumeng and Saltiel, 2011). Leptin normally acts as an endocrine hormone on the hypothalamus to regulate food intake and energy expenditure (Tilg and Moschen, 2006; Musi and Guardado-Mendoza, 2014; Martínez-Uña et al., 2020). Leptin is also a pro-inflammatory cytokine that is overexpressed during meta-inflammation, causing a general inflammatory state, such as in the liver, where it promotes the accumulation of lipids. We observed that Leptin is overexpressed in HFD mouse livers, whereas its expression was downregulated in HFD + *mol*-miR pool livers. Another important pro-inflammatory adipokine is Resistin, which is secreted by adipocytes and macrophages, and is thought to be a link between obesity and type 2 diabetes. Resistin increases LDL production in human hepatocytes, degrades LDL receptors, and reduces the ability of the liver to clear LDL (Tilg and Moschen, 2006). Resistin strongly upregulates the expression of other pro-inflammatory cytokines, such as TNF and IL-6 in human peripheral blood mononuclear cells (Tilg and Moschen, 2006). Accordingly, a consistent increase in the expression of Resistin was observed in HFD mice, whereas its level decreased in HFD + *mol*-miR pool mice. Interestingly, Resistin was downregulated in HepG2 cells treated with the *mol*-miR pool, compared with untreated controls. TNF-alpha was also downregulated and this can be considered as a possible effect of both downregulation of Resistin and direct modulation of *mol-*miRs. Moreover, many genes belonging to families known to be involved in lipid metabolism were upregulated by HFD. These included, GATA binding proteins (GATA), Krüppel-like factors (KLFs), Peroxisome Proliferator Activator Receptors (PPARs), and the wingless-related MMTV integration site (WNTs) (Rosen and MacDougald, 2006). KLFs are a large family of transcription factors involved in processes such as regulation of infection (Minutolo et al., 2014), differentiation, proliferation, and apoptosis (McConnell and Yang, 2010). Interestingly, a substantial increase in KLF2 gene expression was found in HFD mouse. KLF2 has indeed been linked to steatosis and triglyceride accumulation in the liver (Chen et al., 2014; Li et al., 2020). Interestingly, all of the genes that were upregulated in HFD mice, were downregulated in HFD + *mol*-miR pool mice. Another gene involved in the regulation of lipid metabolism is INSR, a transmembrane insulin receptor. Binding of insulin or other ligands to this receptor activates the insulin signalling pathway, which regulates uptake and release of glucose and the synthesis and storage of carbohydrates, lipids, and proteins (Payankaulam et al., 2019). Mutations in the INSR gene underlie inherited syndromes of severe insulin resistance. We found that INSR is overexpressed in the livers of HFD mice whereas a decrease occurs in the HFD group treated with *mol-*miRs. Downregulation is also observed in MOES-treated HepG2 cells. Alteration of lipid metabolism is a crucial mechanism in the development of HCC, as lipid accumulation in hepatocytes leads to cell transformation (Elmore, 2007; Baffy et al., 2012; Gu et al., 2016; Saitta et al., 2019). Indeed, genes related to tumorigenesis were upregulated in HFD mice and downregulated in HFD + *mol*-miR pool mice. Along this line of evidence, previous work suggested the ability of plant miRNAs to restore epithelial–mesenchymal transition (EMT), a mechanism associated with cancer, and lipid accumulation in HepG2 cells (Minutolo et al., 2018). In summary, using human and mouse models we show that MOES regulate lipid metabolism due to the presence of *mol*-miRs in the extract (Supplementary Table S4). In view of these results, *mol*-miRs can be considered as bioactive components of MOES; however, confirmation of this hypothesis requires further in-depth studies. Nevertheless, our results pave the way for a new therapeutic approach based on plant miRNAs for the treatment of diseases such as obesity. These miRNAs can be used as dietary supplements to complement therapeutic treatments and limit their side effects.

## Data Availability

The datasets analysed for this study can be found in the Geo Repository http://www.ncbi.nlm.nih.gov/geo. Accession number: GSE125344 (human), GSE125346 (mouse).
