# Supplementary material for: Exogenous miRNAs from Moringa oleifera Lam. recover a dysregulated lipid metabolism
Source: Front Mol Biosci. 2022 Nov 17;9:1012359. doi: 10.3389/fmolb.2022.1012359 (PMC9715436; doi:10.3389/fmolb.2022.1012359)
Supplement: Supplementary file 1 [file Table1.DOCX]

**Supplementary Table 1** Bioinformatic prediction analysis. 61 human genes modulated by MOES in HepG2 cell line and *mol*-miRs potential targets are reported. The fold changes (FC) of the up‑regulated genes are reported in bold; the FC of the down-regulated genes are reported in italic. The *mol*-miRs are reported with the score

| **Genes** | **Description** | **Pathway** | **HepG2+MOES *vs* HepG2** | ***mol*-miRs** |
| --- | --- | --- | --- | --- |
| Acacb | Acetyl-Coenzyme A carboxylase beta | Pro-Adipogenesis | **4,4146** | miR395d (0.92) |
| Adig | Adipogenin | Adipokines | *0,0169* |  |
| Adipoq | Adiponectin, C1Q and collagen domain | Adipokines | *0,0406* |  |
| Adrb2 | Adrenergic receptor, beta 2 | Anti-Adipogenesis | *0,0942* |  |
| Agt | Angiotensinogen | Hormones | *0,4739* | miR160h (0.65), miR395d (0.96), miR482b (0.97) |
| Angpt2 | Angiopoietin 2 | Hormones | *0,2574* |  |
| Bmp4 | Bone morphogenetic protein 4 | Inflammation and TGFbeta | *0,4481* |  |
| Ccnd1 | Cyclin-dependent kinase inhi 1A (P21) | Pro-Adipogenesis | *0,4365* |  |
| Cdkn1a | Cyclin-dependent kinase inhibitor 1A (P21) | Tumorigenesis | *0,4401* | miR395d (0.93), miR160h (0.96), miR166 (0.97) |
| Cebpd | CCAAT/enhabind prot(C/EBP), delta | Pro-Adipogenesis | **5,5711** | miR482b (0.94) |
| Cfd | Complement factor D (adipsin) | Adipokines | *0,2981* | miR160h (0.95) |
| Creb1 | CAMP responsive element binding protein 1 | Pro-Brown Adipose Tissue | **3,5485** |  |
| Dio2 | Deiodinase, iodothyronine, type II | Pro-Brown Adipose Tissue | 2,3686 | miR166 (0.96), miR396a (0.99) |
| Egr2 | Early growth response 2 | Pro-White Adipose Tissue | *0,0089* | miR166 (0.60), miR159c (0.82) |
| Fabp4 | Fatty acid binding protein 4, adipocyte | Pro-Adipogenesis | *0,235* |  |
| Fasn | Fatty acid synthase | Pro-Adipogenesis/PPAR Gamma | *0,0759* |  |
| Fgf2 | Fibroblast growth factor 2 | Pro-Adipogenesis | *0,2392* |  |
| Foxo1 | Fibroblast growth factor 10 | Pro-White Adipose Tissue | *0,2231* |  |
| Foxc2 | Forkhead box O1 | Anti-Adipogenesis | *0,3729* |  |
| Gata3 | GATA binding protein 3 | Anti-White Adipose Tissue | *0,1408* |  |
| Hes1 | Hairy and enhancer of split 1 (Drosophila) | Anti-Adipogenesis | *0,1744* |  |
| Insr | Insulin receptor | Pro-Brown Adipose Tissue | *0,3864* | miR396a (0.85), miR482b (0.98) |
| Irs1 | Insulin receptor substrate 1 | Beta-Oxidation | *0,2649* |  |
| Irs2 | Insulin receptor substrate 2 | Pro-Adipogenesis | *0,0985* |  |
| Jun | Jun oncogene | Tumorigenesis/p53 | *0,0361* |  |
| Klf2 | Kruppel-like factor 2 (lung) | Anti-White Adipose Tissue | *0,4797* | miR2118a (0.87), miR482b (0.96), miR166 (0.97) |
| Klf15 | Kruppel-like factor 15 | Pro-White Adipose Tissue | *0,2501* |  |
| Lep | Leptin | Adipokines | **3,7469** | miR159c (0.95), miR482b (0.96), miR160h (0.96), miR166 (0.98) |
| Lipe | Lipase, hormone sensitive | Enzymes | *0,4805* |  |
| Lmna | Lamin A | Pro-Adipogenesis | *0,1833* |  |
| Lpl | Lipoprotein lipase | Enzymes | *0,2644* | miR160h (0.61) |
| Lrp5 | Low density lipoprotein receptor-related protein 5 | Anti-Adipogenesis | *0,1586* |  |
| Mapk14 | Mitogen-activated protein kinase 14 | Pro-Brown Adipose Tissue | *0,1788* | miR393a (0.92), miR396a (0.98) |
| Ncoa2 | Nuclear receptor coactivator 2 | Anti-Adipogenesis | *0,2628* |  |
| Nr0b2 | Nuclear receptor subfamily 0, group B, member 2 | Anti-Brown Adipose Tissue | *0,1065* |  |
| Nr1h3 | Nuclear receptor subfamily 1, group H, member 3 | Anti-Brown Adipose Tissue | *0,185* |  |
| Nrf1 | Nuclear respiratory factor 1 | Pro-Brown Adipose Tissue | *0,2898* | miR160h (0.94), miR396a (0.97), miR482b (0.98) |
| Ppara | Peroxisome proliferator receptor alpha | Beta-Oxidation | *0,0448* | miR393a (0.75), miR160h (0.96), miR166 (0.96) |
| Pparg | Peroxisome proliferator activated receptor gamma | Pro-Adipogenesis | *0,0279* |  |
| Ppargc1a | Peroxisome prol. activated rec., gamma, coact. 1 alpha | Pro-Brown Adipose Tissue | *0,0218* |  |
| Ppargc1b | Peroxisome prol. activated rec., gamma, coact. 1 beta | Pro-Brown Adipose Tissue | *0,0915* |  |
| Prdm16 | PR domain containing 16 | Pro-Brown Adipose Tissue | *0,4436* |  |
| Rb1 | Retinoblastoma 1 | Anti-Brown Adipose Tissue | *0,1757* | miR160h (0.86) |
| Retn | Resistin | Adipokines | *0,3856* |  |
| Rxra | Retinoid X receptor alpha | Cholesterol Metabolism & Transport | *0,0834* | miR160h (0.97), miR166 (0.98) |
| Sfrp1 | Secreted frizzled-related protein 1 | Pro-Adipogenesis | *0,169* | miR160h (0.84), miR166 (0.84), miR482b (0.97) |
| Shh | Sonic hedgehog | Tumorigenesis | *0,143* | miR159c (0.95), miR482b (0.98) |
| Sirt1 | Sirtuin 1 (S. cerevisiae) | Tumorigenesis/p53 | *0,279* |  |
| Sirt2 | Sirtuin 2 (S. cerevisiae) | Anti-Adipogenesis | *0,3178* |  |
| Sirt3 | Sirtuin 3 (S. cerevisiae) | Pro-Brown Adipose Tissue | *0,054* |  |
| Slc2a4 | Solute carrier family 2, member 4 | Pro-Adipogenesis | *0,3371* |  |
| Srebf1 | Sterol regulatory element binding transcription factor 1 | PPAR Gamma Targets | *0,1713* |  |
| Taz | Tafazzin | Anti-Adipogenesis | *0,1196* |  |
| Tcf7l2 | Transcription factor 7-like 2, T-cell specific, HMG-box | Anti-Adipogenesis | *0,1071* |  |
| Tsc22d3 | TSC22 domain family, member 3 | Anti-Adipogenesis | *0,0098* |  |
| Twist1 | Twist homolog 1 (Drosophila) | Tumorigenesis | *0,0187* | miR160h (0.80) |
| Ucp1 | Uncoupling protein 1 (mitochondrial, proton carrier) | Pro-Brown Adipose Tissue | *0,1784* |  |
| Vdr | Vitamin D receptor | Tumorigenesis | *0,2951* | miR160h (0.71), miR166 (0.97) |
| Wnt1 | Wingless-related MMTV integration site 1 | Anti-Adipogenesis | *0,3016* |  |
| Wnt3a | Wingless-related MMTV integration site 3A | Tumorigenesis | *0,0139* | miR171b (0.87), miR166 (0.92), miR482b (0.94), miR2118a (0.96), miR160h (0.98) |
| Wnt10b | Wingless related MMTV integration site 10b | Tumorigenesis | *0,167* | miR166 (0.62), miR167f-3p (0.88), miR395d (0.96), miR482b (0.96), miR2118a (0.96), miR160h (0.98) |
