# Supplementary material for: Exogenous miRNAs from Moringa oleifera Lam. recover a dysregulated lipid metabolism
Source: Front Mol Biosci. 2022 Nov 17;9:1012359. doi: 10.3389/fmolb.2022.1012359 (PMC9715436; doi:10.3389/fmolb.2022.1012359)
Supplement: Supplementary file 2 [file Table2.DOCX]

**Supplementary Table 2** Human and murine target genes potentially regulated by *mol*-miRs. The fold change (FC) of up-regulated (bold) and down-regulated (italic) genes is shown. For each *mol*‑miR, the binding energy, shown as score, is reported. Human and murine genes, which appear to be potential targets of *mol*-miRs, are highlighted in gray

| **Genes** | **HepG2+MOES *vs* HepG2** | ***mol*-miRs predicted for human genes** | ***mol*-miRs predicted for murine genes** |
| --- | --- | --- | --- |
| Acacb | **4,4146** | miR395d (0.92) |  |
| Adig | *0,0169* |  | miR160h (0,96), miR159c (0,96) |
| Adipoq | *0,0406* |  | miR160h (0,96), miR156e (0,92) |
| Adrb2 | *0,0942* |  |  |
| Agt | *0,4739* | miR160h (0.65), miR395d (0.96), miR482b (0.97) | miR160h (0,90), miR159c (0,90), miR167f-3p(0,96), miR397a(0,68) |
| Angpt2 | *0,2574* |  |  |
| Bmp4 | *0,4481* |  |  |
| Ccnd1 | *0,4365* |  | miR167f-3p (0,93), miR160h (0,91), miR166(0,92), miR397a (0,89) |
| Cdkn1a | *0,4401* | miR395d (0.93), miR160h (0.96), miR166 (0.97) | miR160h (0,96) |
| Cebpd | **5,5711** | miR482b (0.94), |  |
| Cfd | *0,2981* | miR160h (0.95) | miR160h (0,97) |
| Creb1 | **3,5485** |  |  |
| Dio2 | **2,3686** | miR166 (0.96), miR396a (0.99) | miR159c (0,94), miR156e (0,94), miR166(0,87), |
| Egr2 | *0,0089* | miR166 (0.60), miR159c (0.82) | miR167f-3p (0,95), miR159c ( 0,94) |
| Fabp4 | *0,235* |  | miR166 (0,96), miR393a (0,96) |
| Fasn | *0,0759* |  | miR2118a (0,91), miR482b (0,67) |
| Fgf2 | *0,2392* |  | miR160h (0,95), miR166(0,92), miR395d (0,85) |
| Foxo1 | *0,2231* |  |  |
| Foxc2 | *0,3729* |  | miR397a (0,96) |
| Gata3 | *0,1408* | - | miR395d (0,95) |
| Hes1 | *0,1744* |  | miR160h (0,96), miR159c (0,90) |
| Insr | *0,3864* | miR396a (0.85), miR482b (0.98) | miR395d (0,90) |
| Irs1 | *0,2649* |  | miR160h (0,95), miR482b (0,85) |
| Irs2 | *0,0985* |  |  |
| Jun | *0,0361* |  | miR160h (0,96), miR858b (0,93), miR159c (0,90), miR166 (0,90) |
| Klf2 | *0,4797* | miR2118a (0.87), miR482b (0.96), miR166 (0.97) | miR166 (0,97), miR160h (0,88) |
| Klf15 | *0,2501* |  | miR160h (0,95) |
| Lep | **3,7469** | miR159c (0.95), miR482b (0.96), miR160h (0.96), miR166 (0.98) | miR395d (0,95), miR159c (0,91), miR160h (0,92) |
| Lipe | *0,4805* |  |  |
| Lmna | *0,1833* |  |  |
| Lpl | *0,2644* | miR160h (0.61) | miR167f-3p (0,65), |
| Lrp5 | *0,1586* |  |  |
| Mapk14 | *0,1788* | miR393a (0.92), miR396a (0.98) | miR159c (0,93) |
| Ncoa2 | *0,2628* |  |  |
| Nr0b2 | *0,1065* |  | miR160h (0,96), miR395d (0,72) |
| Nr1h3 | *0,185* |  | miR482b (0,96), miR160h (0,88), |
| Nrf1 | *0,2898* | miR160h (0.94), miR396a (0.97), miR482b (0.98) | miR167f-3p (0,85) |
| Ppara | *0,0448* | miR393a (0.75), miR160h (0.96), miR166 (0.96) | miR397a (0,96), miR482b (0,94), miR160h (0,87) |
| Pparg | *0,0279* |  |  |
| Ppargc1a | *0,0218* |  |  |
| Ppargc1b | *0,0915* |  | miR159c (0,90) |
| Prdm16 | *0,4436* |  |  |
| Rb1 | *0,1757* | miR160h (0.86) | miR159c (0,61) |
| Retn | *0,3856* |  | miR396a (0,93), miR166 (0,93), miR167f-3p (0,85), miR160h (0,91) |
| Rxra | *0,0834* | miR160h (0.97), miR166 (0.98) | miR160h (0,93), miR159c (0,93), miR398a-5p (0,61), miR166 (0,61) |
| Sfrp1 | *0,169* | miR160h (0.84), miR166 (0.84), miR482b (0.97) | miR160h (0,92), miR166 (0,92), |
| Shh | *0,143* | miR159c (0.95), miR482b (0.98), | miR858b (0,96), miR167f-3p (0,72) |
| Sirt1 | *0,279* |  | miR858b (0,92) |
| Sirt2 | *0,3178* |  | miR395d (0,93) |
| Sirt3 | *0,054* |  |  |
| Slc2a4 | *0,3371* |  |  |
| Srebf1 | *0,1713* |  | miR398a-5p (0,92) |
| Taz | *0,1196* |  | miR396a (0,64) |
| Tcf7l2 | *0,1071* |  |  |
| Tsc22d3 | *0,0098* |  |  |
| Twist1 | *0,0187* | miR160h (0.80) |  |
| Ucp1 | *0,1784* |  | miR159c (0,95), miR160h (0,95) |
| Vdr | *0,2951* | miR160h (0.71), miR166 (0.97) | miR160h (0,91), miR398a-5p (0,91), miR166 (0,63) |
| Wnt1 | *0,3016* |  | miR482b (0,69), miR395d (0,65) |
| Wnt3a | *0,0139* | miR171b (0.87), miR166 (0.92), miR482b (0.94), miR2118a (0.96), miR160h (0.98) | miR159c (0,93), miR395d (0,85) |
| Wnt10b | *0,167* | miR166 (0.62), miR167f-3p (0.88), miR395d (0.96), miR482b (0.96), miR2118a (0.96), miR160h (0.98) | miR858b (0,96), miR167f-3p (0,94), miR160h (0,91) |
