# Supplementary material for: Exogenous miRNAs from Moringa oleifera Lam. recover a dysregulated lipid metabolism
Source: Front Mol Biosci. 2022 Nov 17;9:1012359. doi: 10.3389/fmolb.2022.1012359 (PMC9715436; doi:10.3389/fmolb.2022.1012359)
Supplement: Supplementary file 3 [file Table3.DOCX]

**Supplementary Table 3** Hepatic gene regulation in mice. Genes modulated by the mol-sR pool reported by function, considering the QIAGEN gene list for Adipogenesis and Fatty Liver. The fold changes (FC) of the up-regulated genes are reported in bold; the FC of the down-regulated genes are reported in italic

| **Ref. Seq.** | **Gene** | | **ND+*mol‑*miRs *vs*  ND** | | **HFD  *vs*  ND** | | **HFD+*mol‑*miRs  *vs*  HFD** | | **HFD+*mol‑*miRs *vs*  ND** |
| --- | --- | --- | --- | --- | --- | --- | --- | --- | --- |
| Regulation of Adipogenesis | | | | | | | | | |
| Adipokines | | | | | | | | | |
| NM_009605 | Adipoq | | **2,6123** | | 6,7225 | | *0,3577* | | **2,4049** |
| NM_013459 | Cfd | | 1,0551 | | **12,9511** | | *0,1466* | | 1,8987 |
| NM_008493 | Lep | | 0,8738 | | **55,8699** | | *0,0338* | | 1,8895 |
| NM_022984 | Retn | | 1,5035 | | **66,7641** | | *0,0143* | | 0,9526 |
| Hormones | | | | | | | | | |
| NM_007428 | Agt | | 1,4293 | | **6,9692** | | 0,5274 | | **3,6757** |
| Lipases | |  | |  | |  | |  | |
| NM_008509 | Lpl | | **2,4105** | | **4,1785** | | *0,4681* | | 1,9561 |
| Pro-Adipogenesis | | | | | | | | | |
| NM_007679 | Cebpd | | 1,9444 | | **26,1547** | | *0,0584* | | 1,5273 |
| NM_010051 | Dkk1 | | 1,8952 | | **84,098** | | *0,0406* | | **3,4105** |
| NM_007891 | E2f1 | | 1,2306 | | **3,8718** | | *0,3716* | | 1,4389 |
| NM_024406 | Fabp4 | | 1,7223 | | *0,3475* | | **2,7549** | | 0,9573 |
| NM_013519 | Fgf2 | | 0,8187 | | **24,8298** | | *0,0297* | | 0,7366 |
| NM_013834 | Sfrp1 | | 1,9123 | | **36,6566** | | *0,0755* | | **2,7664** |
| NM_018780 | Sfrp5 | | **2,2444** | | **66,7641** | | *0,039* | | **2,6063** |
| Anti-Adipogenesis | | | | | | | | | |
| NM_010052 | Dlk1 | | **3,905** | | **112,9077** | | *0,0311* | | **3,5137** |
| NM_009822 | Runx1t1 | | 0,6922 | | **23,7359** | | *0,0795* | | 1,8882 |
| Pro-White Adipose Tissue | | | | | | | | | |
| NM_010118 | Egr2 | | **4,2115** | | **14,97** | | *0,1266* | | 1,8947 |
| NM_008006 | Fgf10 | | 1,8691 | | **77,6546** | | *0,0217* | | 1,6818 |
| NM_010637 | Klf4 | | 1,889 | | **14,2907** | | *0,0891* | | 1,2728 |
| Anti-White Adipose Tissue | | | | | | | | | |
| NM_008090 | Gata2 | | **2,0368** | | **52,3457** | | *0,035* | | 1,8327 |
| NM_008091 | Gata3 | | 0,567 | | **44,2008** | | *0,0231* | | 1,0203 |
| NM_008452 | Klf2 | | **2,215** | | **38,4524** | | *0,0328* | | 1,2605 |
| Pro-Brown Adipose Tissue | | | | | | | | | |
| NM_010050 | Dio2 | | 0,8823 | | **47,5048** | | *0,0167* | | 0,7939 |
| NM_008090 | Foxc2 | | 1,0772 | | *0,0819* | | 11,8351 | | 0,9693 |
| NM_011951 | Mapk14 | | **2,3955** | | **3,8504** | | *0,2774* | | 1,0681 |
| NM_010938 | Nrf1 | | 1,5826 | | **3,7659** | | *0,4659* | | 1,7544 |
| NM_009463 | Ucp1 | | 0,8835 | | *0,0992* | | **17,3276** | | 1,7195 |
| Anti-Brown Adipose Tissue | | | | | | | | | |
| NM_009029 | Rb1 | | 1,8498 | | **6,3467** | | *0,355* | | **2,2532** |
| Metabolic Pathways | |  | |  | |  | |  | |
| Beta-Oxidation | | | | | | | | | |
| NM_010570 | Irs1 | | 1,2669 | | *0,3989* | | **7,1801** | | **2,8639** |
| NM_011144 | Ppara | | 0,8013 | | **2,3784** | | *0,2655* | | 0,6316 |
| Cholesterol Metabolism & Transport | | | | | | | | | |
| NM_011145 | Ppard | | 0,6477 | | **3,5578** | | *0,3281* | | 1,1672 |
| NM_011305 | Rxra | | 0,9287 | | **7,3973** | | *0,1665* | | 1,232 |
| Tumorigenesis | | | | | | | | | |
| NM_009733 | Axin1 | | 1,1434 | | *0,2672* | | **2,6226** | | 0,7008 |
| NM_007553 | Bmp2 | | 1,4136 | | **27,7228** | | *0,0459* | | 1,2719 |
| NM_007557 | Bmp7 | | 1,3098 | | **6,9596** | | *0,1693* | | 1,1785 |
| NM_009870 | Cdk4 | | 1,0668 | | **2,9019** | | *0,2936* | | 0,852 |
| NM_007669 | Cdkn1a | | 1,9084 | | **8,0724** | | *0,2657* | | **2,145** |
| NM_009170 | Shh | | *0,4973* | | **11,0655** | | *0,1472* | | 1,629 |
| NM_009271 | Src | | 0,7097 | | **5,3369** | | *0,3244* | | 1,7315 |
| NM_011658 | Twist1 | | 0,93 | | **4,1728** | | *0,4081* | | 1,7029 |
| NM_009504 | Vdr | | 1,2873 | | **61,7343** | | *0,0239* | | 1,4763 |
| NM_009522 | Wnt3a | | **2,3757** | | **47,4061** | | *0,0329* | | 1,5583 |
| NM_009524 | Wnt5a | | 1,2187 | | **12,295** | | *0,1222* | | 1,5021 |
| NM_011718 | Wnt10b | | 1,643 | | **39,7257** | | *0,062* | | **2,464** |
