# Supplementary material for: Exogenous miRNAs from Moringa oleifera Lam. recover a dysregulated lipid metabolism
Source: Front Mol Biosci. 2022 Nov 17;9:1012359. doi: 10.3389/fmolb.2022.1012359 (PMC9715436; doi:10.3389/fmolb.2022.1012359)
Supplement: Supplementary file 4 [file Table4.DOCX]

**Supplementary Table 4** Genes modulated by MOES in the HepG2 cell line and by *mol*-miR pool in pre-obese mice The fold changes (FC) of the up-regulated genes are reported in bold; the FC of the down-regulated genes are reported in italic

| **Gene name** | **HFD *vs* ND** | **HFD + *mol‑*miR *vs* ND** | **HepG2 + MOES *vs* HepG2** | ***mol*-miR target human genes** | ***mol*-miR target  murine genes** |
| --- | --- | --- | --- | --- | --- |
| Agt | 6,9692 | 3,6757 | *0,4739* | miR160h (0.65), miR395d (0.96), miR482b (0.97) | miR160h(0,90), miR159c(0,90), miR167f-3p(0,96), miR397a(0,68) |
| Cdkn1a | 8,0724 | 2,145 | *0,4401* | miR395d (0.93), miR160h (0.96), miR166 (0.97) | miR160h(0,96) |
| Cfd | 12,9511 | 1,8987 | *0,2981* | miR160h (0.95) | miR160h(0,97) |
| Dio2 | 47,5048 | 0,7939 | 2,3686 | miR166 (0.96), miR396a (0.99) | miR159c(0,94), miR156e(0,94), miR166(0,87), |
| Egr2 | 14,97 | 1,8947 | *0,0089* | miR166 (0.60), miR159c (0.82) | miR167f-3p(0,95), miR159c(0,94) |
| Insr | 4,9041 | 2,5193 | *0,3864* | miR396a (0.85), miR482b (0.98) | miR395d(0,90) |
| Klf2 | 38,4524 | 1,2605 | *0,4797* | miR2118a (0.87), miR482b (0.96), miR166 (0.97) | miR166(0,97), miR160h(0,88) |
| Lep | 55,8699 | 1,8895 | 3,7469 | miR159c (0.95), miR482b (0.96), miR160h (0.96), miR166 (0.98) | miR395d(0,95), miR159c(0,91), miR160h(0,92) |
| Lpl | 4,1785 | 1,9561 | *0,2644* | miR160h (0.61) | miR167f-3p(0,65), |
| Mapk14 | 3,8504 | 1,0681 | *0,1788* | miR393a (0.92), miR396a (0.98) | miR159c(0,93) |
| Nrf1 | 3,7659 | 1,7544 | *0,2898* | miR160h (0.94), miR396a (0.97), miR482b (0.98) | miR167f-3p(0,85) |
| Ppara | 2,3784 | 0,6316 | *0,0448* | miR393a (0.75), miR160h (0.96), miR166 (0.96) | miR397a(0,96), miR482b(0,94), miR160h(0,87) |
| Rb1 | 6,3467 | 2,2532 | *0,1757* | miR160h (0.86) | miR159c(0,61) |
| Rxra | 7,3973 | 1,232 | *0,0834* | miR160h (0.97), miR166 (0.98) | miR160h(0,93), miR159c(0,93), miR398a-5p(0,61), miR166(0,61) |
| Sfrp1 | 36,6566 | 2,7664 | *0,169* | miR160h (0.84), miR166 (0.84), miR482b (0.97) | miR160h(0,92), miR166(0,92), |
| Shh | 11,0655 | 1,629 | *0,143* | miR159c (0.95), miR482b (0.98), | miR858b(0,96), miR167f-3p(0,72) |
| Vdr | 61,7343 | 1,4763 | *0,2951* | miR160h (0.71), miR166 (0.97) | miR160h(0,91), miR398a-5p(0,91), miR166(0,63) |
| Wnt3a | 47,4061 | 1,5583 | *0,0139* | miR171b (0.87), miR166 (0.92), miR482b (0.94), miR2118a (0.96), miR160h (0.98) | miR159c(0,93), miR395d(0,85) |
| Wnt10b | 39,7257 | 2,464 | *0,167* | miR166 (0.62), miR167f-3p (0.88), miR395d (0.96), miR482b (0.96), miR2118a (0.96), miR160h (0.98) | miR858b(0,96), miR167f-3p(0,94), miR160h(0,91) |
