# Supplementary material for: Exogenous miRNAs from Moringa oleifera Lam. recover a dysregulated lipid metabolism
Source: Front Mol Biosci. 2022 Nov 17;9:1012359. doi: 10.3389/fmolb.2022.1012359 (PMC9715436; doi:10.3389/fmolb.2022.1012359)
Supplement: Supplementary file 5 [file DataSheet1.docx]

Supplementary Material

# Experimental strategy

A graphical representation of the experimental design is presented in Supplementary Figure 1. *In vitro* studies: HepG2 hepatic tumor cells were treated with different concentrations of MOES for 72 h, and cell viability rates, apoptosis, and intracellular accumulation of lipid droplets were measured. Total RNA was extracted from HepG2 cells, and the expression of human lipid metabolism genes was assessed, and the data were explored by bioinformatics analysis to define the potential interactions between *mol*-miRs and human genes. Subsequently, HepG2 cells were transfected with a pool of *mol*-miR, and the same variables observed in the MOES cell treatments were evaluated (Supplementary Figure 1A). Before moving on to the *in vivo* study, the *Mus musculus* mRNA targets of *mol*-miRs were assessed. To investigate the effects of *mol*-miRs on lipid metabolism gene expression, a study was performed using a pre-obesity mouse model (Supplementary Figure 1B). For sample size determination, a pre-hoc statistical analysis was performed and a sample size of n=3 mice in each group was estimated. C57BL/6J mice were randomly divided into two groups (six mice/group): mice fed a normal-calorie diet (ND) and those fed a high-fat diet (HFD). Three mice from each group, were treated with the *mol*-miR pool (ND + *mol-*miRs and HFD + *mol-*miRs) by oral gavage every two days, and dietary treatments were maintained for five weeks. Mouse body weight was measured every week from the start of treatment, and plasma glucose and total cholesterol levels were measured at the end of treatment. Mice were sacrificed, the livers were explanted and weighed, and histological analysis was performed to study lipid accumulation (Supplementary Figure 1C). RNA was extracted from mouse livers to study the expression of lipid metabolism-related genes and to detect the presence of *mol*-miRs.

# Supplementary Figures

Supplementary Figure 1 Graphical representation of the experimental design. This scheme schematically depicts the study conducted with (A) MOES and *mol*-miRs *in vitro* and (B) *in vivo*. (A) HepG2 cells were treated for 72 hours with MOES or *mol*-miRs, and the variables analyzed were reported. (B) Mice were treated three times per week (t.i.w.) and were sacrificed at 5th week to analyze the parameters indicated in (C). Created with BioRender.com.

Supplementary Figure 2 Representative gating strategy used for the analysis of BODYPI (BPI) assay by Flow cytometry. For the analysis of the lipid accumulation, the HepG2 cells were stained with BPI. In the live cell gate (panel A: HepG2+Vehicle (HF); panel B: HepG2 + *mol*‑miR pool), 20000 events were recorded for each sample. The BPI analysis has been reported on a dot plot on the FL1 fluorescence *vs* SSC-A (panel A-E).

Supplementary Figure 3 The Heat Map show the expression of upregulated (red) and downregulated (green) genes in livers of mice subjected to HFD with respect to ND group, and relative table that provides the fold change data used for the Heat Map.

Supplementary Figure 4 The Heat Map show the expression of upregulated (red) and downregulated (green) genes in livers of mice subjected to HFD + *mol*-miR pool with respect to ND group, and relative table that provides the fold change data used for the Heat Map.

Supplementary Figure 5 The Heat Map show the expression of upregulated (red) and downregulated (green) genes in livers of mice subjected to HDF + *mol*-miR pool with respect to HFD group, and relative table that provides the fold change data used for the Heat Map.

Supplementary Figure 6 Liver dot plot array profile of ND *vs* ND + *mol*-miR pool. Transcriptional levels of specific genes are shown as fold change. Values >1.50 or <0.5 were considered significantly upregulated (red dots) and downregulated (green dots), respectively. The Heat Map show the expression of upregulated (red) and downregulated (green) genes in livers of mice subjected to ND + *mol*-miR pool with respect to ND group, and relative table that provides the fold change data used for the Heat Map

# Supplementary Tables

**Supplementary Table 1** Bioinformatic prediction analysis. 61 human genes modulated by MOES in HepG2 cell line and *mol*-miRs potential targets are reported. The fold changes (FC) of the upregulated genes are reported in bold; the FC of the downregulated genes are reported in italic. The *mol*-miRs are reported with the score

| **Genes** | **Description** | **Pathway** | **HepG2+MOES *vs* HepG2** | ***mol*-miRs** |
| --- | --- | --- | --- | --- |
| Acacb | Acetyl-Coenzyme A carboxylase beta | Pro-Adipogenesis | **4,4146** | miR395d (0.92) |
| Adig | Adipogenin | Adipokines | *0,0169* |  |
| Adipoq | Adiponectin, C1Q and collagen domain | Adipokines | *0,0406* |  |
| Adrb2 | Adrenergic receptor, beta 2 | Anti-Adipogenesis | *0,0942* |  |
| Agt | Angiotensinogen | Hormones | *0,4739* | miR160h (0.65), miR395d (0.96), miR482b (0.97) |
| Angpt2 | Angiopoietin 2 | Hormones | *0,2574* |  |
| Bmp4 | Bone morphogenetic protein 4 | Inflammation and TGFbeta | *0,4481* |  |
| Ccnd1 | Cyclin-dependent kinase inhi 1A (P21) | Pro-Adipogenesis | *0,4365* |  |
| Cdkn1a | Cyclin-dependent kinase inhibitor 1A (P21) | Tumorigenesis | *0,4401* | miR395d (0.93), miR160h (0.96), miR166 (0.97) |
| Cebpd | CCAAT/enhabind prot(C/EBP), delta | Pro-Adipogenesis | **5,5711** | miR482b (0.94) |
| Cfd | Complement factor D (adipsin) | Adipokines | *0,2981* | miR160h (0.95) |
| Creb1 | CAMP responsive element binding protein 1 | Pro-Brown Adipose Tissue | **3,5485** |  |
| Dio2 | Deiodinase, iodothyronine, type II | Pro-Brown Adipose Tissue | 2,3686 | miR166 (0.96), miR396a (0.99) |
| Egr2 | Early growth response 2 | Pro-White Adipose Tissue | *0,0089* | miR166 (0.60), miR159c (0.82) |
| Fabp4 | Fatty acid binding protein 4, adipocyte | Pro-Adipogenesis | *0,235* |  |
| Fasn | Fatty acid synthase | Pro-Adipogenesis/PPAR Gamma | *0,0759* |  |
| Fgf2 | Fibroblast growth factor 2 | Pro-Adipogenesis | *0,2392* |  |
| Foxo1 | Fibroblast growth factor 10 | Pro-White Adipose Tissue | *0,2231* |  |
| Foxc2 | Forkhead box O1 | Anti-Adipogenesis | *0,3729* |  |
| Gata3 | GATA binding protein 3 | Anti-White Adipose Tissue | *0,1408* |  |
| Hes1 | Hairy and enhancer of split 1 (Drosophila) | Anti-Adipogenesis | *0,1744* |  |
| Insr | Insulin receptor | Pro-Brown Adipose Tissue | *0,3864* | miR396a (0.85), miR482b (0.98) |
| Irs1 | Insulin receptor substrate 1 | Beta-Oxidation | *0,2649* |  |
| Irs2 | Insulin receptor substrate 2 | Pro-Adipogenesis | *0,0985* |  |
| Jun | Jun oncogene | Tumorigenesis/p53 | *0,0361* |  |
| Klf2 | Kruppel-like factor 2 (lung) | Anti-White Adipose Tissue | *0,4797* | miR2118a (0.87), miR482b (0.96), miR166 (0.97) |
| Klf15 | Kruppel-like factor 15 | Pro-White Adipose Tissue | *0,2501* |  |
| Lep | Leptin | Adipokines | **3,7469** | miR159c (0.95), miR482b (0.96), miR160h (0.96), miR166 (0.98) |
| Lipe | Lipase, hormone sensitive | Enzymes | *0,4805* |  |
| Lmna | Lamin A | Pro-Adipogenesis | *0,1833* |  |
| Lpl | Lipoprotein lipase | Enzymes | *0,2644* | miR160h (0.61) |
| Lrp5 | Low density lipoprotein receptor-related protein 5 | Anti-Adipogenesis | *0,1586* |  |
| Mapk14 | Mitogen-activated protein kinase 14 | Pro-Brown Adipose Tissue | *0,1788* | miR393a (0.92), miR396a (0.98) |
| Ncoa2 | Nuclear receptor coactivator 2 | Anti-Adipogenesis | *0,2628* |  |
| Nr0b2 | Nuclear receptor subfamily 0, group B, member 2 | Anti-Brown Adipose Tissue | *0,1065* |  |
| Nr1h3 | Nuclear receptor subfamily 1, group H, member 3 | Anti-Brown Adipose Tissue | *0,185* |  |
| Nrf1 | Nuclear respiratory factor 1 | Pro-Brown Adipose Tissue | *0,2898* | miR160h (0.94), miR396a (0.97), miR482b (0.98) |
| Ppara | Peroxisome proliferator receptor alpha | Beta-Oxidation | *0,0448* | miR393a (0.75), miR160h (0.96), miR166 (0.96) |
| Pparg | Peroxisome proliferator activated receptor gamma | Pro-Adipogenesis | *0,0279* |  |
| Ppargc1a | Peroxisome prol. activated rec., gamma, coact. 1 alpha | Pro-Brown Adipose Tissue | *0,0218* |  |
| Ppargc1b | Peroxisome prol. activated rec., gamma, coact. 1 beta | Pro-Brown Adipose Tissue | *0,0915* |  |
| Prdm16 | PR domain containing 16 | Pro-Brown Adipose Tissue | *0,4436* |  |
| Rb1 | Retinoblastoma 1 | Anti-Brown Adipose Tissue | *0,1757* | miR160h (0.86) |
| Retn | Resistin | Adipokines | *0,3856* |  |
| Rxra | Retinoid X receptor alpha | Cholesterol Metabolism & Transport | *0,0834* | miR160h (0.97), miR166 (0.98) |
| Sfrp1 | Secreted frizzled-related protein 1 | Pro-Adipogenesis | *0,169* | miR160h (0.84), miR166 (0.84), miR482b (0.97) |
| Shh | Sonic hedgehog | Tumorigenesis | *0,143* | miR159c (0.95), miR482b (0.98) |
| Sirt1 | Sirtuin 1 (S. cerevisiae) | Tumorigenesis/p53 | *0,279* |  |
| Sirt2 | Sirtuin 2 (S. cerevisiae) | Anti-Adipogenesis | *0,3178* |  |
| Sirt3 | Sirtuin 3 (S. cerevisiae) | Pro-Brown Adipose Tissue | *0,054* |  |
| Slc2a4 | Solute carrier family 2, member 4 | Pro-Adipogenesis | *0,3371* |  |
| Srebf1 | Sterol regulatory element binding transcription factor 1 | PPAR Gamma Targets | *0,1713* |  |
| Taz | Tafazzin | Anti-Adipogenesis | *0,1196* |  |
| Tcf7l2 | Transcription factor 7-like 2, T-cell specific, HMG-box | Anti-Adipogenesis | *0,1071* |  |
| Tsc22d3 | TSC22 domain family, member 3 | Anti-Adipogenesis | *0,0098* |  |
| Twist1 | Twist homolog 1 (Drosophila) | Tumorigenesis | *0,0187* | miR160h (0.80) |
| Ucp1 | Uncoupling protein 1 (mitochondrial, proton carrier) | Pro-Brown Adipose Tissue | *0,1784* |  |
| Vdr | Vitamin D receptor | Tumorigenesis | *0,2951* | miR160h (0.71), miR166 (0.97) |
| Wnt1 | Wingless-related MMTV integration site 1 | Anti-Adipogenesis | *0,3016* |  |
| Wnt3a | Wingless-related MMTV integration site 3A | Tumorigenesis | *0,0139* | miR171b (0.87), miR166 (0.92), miR482b (0.94), miR2118a (0.96), miR160h (0.98) |
| Wnt10b | Wingless related MMTV integration site 10b | Tumorigenesis | *0,167* | miR166 (0.62), miR167f-3p (0.88), miR395d (0.96), miR482b (0.96), miR2118a (0.96), miR160h (0.98) |

**Supplementary Table 2** Human and murine target genes potentially regulated by *mol*-miRs. The fold change (FC) of upregulated (bold) and downregulated (italic) genes is shown. For each *mol*‑miR, the binding energy, shown as score, is reported. Human and murine genes, which appear to be potential targets of *mol*-miRs, are highlighted in gray

| **Genes** | **HepG2+MOES *vs* HepG2** | ***mol*-miRs predicted for human genes** | ***mol*-miRs predicted for murine genes** |
| --- | --- | --- | --- |
| Acacb | **4,4146** | miR395d (0.92) |  |
| Adig | *0,0169* |  | miR160h (0,96), miR159c (0,96) |
| Adipoq | *0,0406* |  | miR160h (0,96), miR156e (0,92) |
| Adrb2 | *0,0942* |  |  |
| Agt | *0,4739* | miR160h (0.65), miR395d (0.96), miR482b (0.97) | miR160h (0,90), miR159c (0,90), miR167f-3p(0,96), miR397a(0,68) |
| Angpt2 | *0,2574* |  |  |
| Bmp4 | *0,4481* |  |  |
| Ccnd1 | *0,4365* |  | miR167f-3p (0,93), miR160h (0,91), miR166(0,92), miR397a (0,89) |
| Cdkn1a | *0,4401* | miR395d (0.93), miR160h (0.96), miR166 (0.97) | miR160h (0,96) |
| Cebpd | **5,5711** | miR482b (0.94), |  |
| Cfd | *0,2981* | miR160h (0.95) | miR160h (0,97) |
| Creb1 | **3,5485** |  |  |
| Dio2 | **2,3686** | miR166 (0.96), miR396a (0.99) | miR159c (0,94), miR156e (0,94), miR166(0,87), |
| Egr2 | *0,0089* | miR166 (0.60), miR159c (0.82) | miR167f-3p (0,95), miR159c ( 0,94) |
| Fabp4 | *0,235* |  | miR166 (0,96), miR393a (0,96) |
| Fasn | *0,0759* |  | miR2118a (0,91), miR482b (0,67) |
| Fgf2 | *0,2392* |  | miR160h (0,95), miR166(0,92), miR395d (0,85) |
| Foxo1 | *0,2231* |  |  |
| Foxc2 | *0,3729* |  | miR397a (0,96) |
| Gata3 | *0,1408* | - | miR395d (0,95) |
| Hes1 | *0,1744* |  | miR160h (0,96), miR159c (0,90) |
| Insr | *0,3864* | miR396a (0.85), miR482b (0.98) | miR395d (0,90) |
| Irs1 | *0,2649* |  | miR160h (0,95), miR482b (0,85) |
| Irs2 | *0,0985* |  |  |
| Jun | *0,0361* |  | miR160h (0,96), miR858b (0,93), miR159c (0,90), miR166 (0,90) |
| Klf2 | *0,4797* | miR2118a (0.87), miR482b (0.96), miR166 (0.97) | miR166 (0,97), miR160h (0,88) |
| Klf15 | *0,2501* |  | miR160h (0,95) |
| Lep | **3,7469** | miR159c (0.95), miR482b (0.96), miR160h (0.96), miR166 (0.98) | miR395d (0,95), miR159c (0,91), miR160h (0,92) |
| Lipe | *0,4805* |  |  |
| Lmna | *0,1833* |  |  |
| Lpl | *0,2644* | miR160h (0.61) | miR167f-3p (0,65), |
| Lrp5 | *0,1586* |  |  |
| Mapk14 | *0,1788* | miR393a (0.92), miR396a (0.98) | miR159c (0,93) |
| Ncoa2 | *0,2628* |  |  |
| Nr0b2 | *0,1065* |  | miR160h (0,96), miR395d (0,72) |
| Nr1h3 | *0,185* |  | miR482b (0,96), miR160h (0,88), |
| Nrf1 | *0,2898* | miR160h (0.94), miR396a (0.97), miR482b (0.98) | miR167f-3p (0,85) |
| Ppara | *0,0448* | miR393a (0.75), miR160h (0.96), miR166 (0.96) | miR397a (0,96), miR482b (0,94), miR160h (0,87) |
| Pparg | *0,0279* |  |  |
| Ppargc1a | *0,0218* |  |  |
| Ppargc1b | *0,0915* |  | miR159c (0,90) |
| Prdm16 | *0,4436* |  |  |
| Rb1 | *0,1757* | miR160h (0.86) | miR159c (0,61) |
| Retn | *0,3856* |  | miR396a (0,93), miR166 (0,93), miR167f-3p (0,85), miR160h (0,91) |
| Rxra | *0,0834* | miR160h (0.97), miR166 (0.98) | miR160h (0,93), miR159c (0,93), miR398a-5p (0,61), miR166 (0,61) |
| Sfrp1 | *0,169* | miR160h (0.84), miR166 (0.84), miR482b (0.97) | miR160h (0,92), miR166 (0,92), |
| Shh | *0,143* | miR159c (0.95), miR482b (0.98), | miR858b (0,96), miR167f-3p (0,72) |
| Sirt1 | *0,279* |  | miR858b (0,92) |
| Sirt2 | *0,3178* |  | miR395d (0,93) |
| Sirt3 | *0,054* |  |  |
| Slc2a4 | *0,3371* |  |  |
| Srebf1 | *0,1713* |  | miR398a-5p (0,92) |
| Taz | *0,1196* |  | miR396a (0,64) |
| Tcf7l2 | *0,1071* |  |  |
| Tsc22d3 | *0,0098* |  |  |
| Twist1 | *0,0187* | miR160h (0.80) |  |
| Ucp1 | *0,1784* |  | miR159c (0,95), miR160h (0,95) |
| Vdr | *0,2951* | miR160h (0.71), miR166 (0.97) | miR160h (0,91), miR398a-5p (0,91), miR166 (0,63) |
| Wnt1 | *0,3016* |  | miR482b (0,69), miR395d (0,65) |
| Wnt3a | *0,0139* | miR171b (0.87), miR166 (0.92), miR482b (0.94), miR2118a (0.96), miR160h (0.98) | miR159c (0,93), miR395d (0,85) |
| Wnt10b | *0,167* | miR166 (0.62), miR167f-3p (0.88), miR395d (0.96), miR482b (0.96), miR2118a (0.96), miR160h (0.98) | miR858b (0,96), miR167f-3p (0,94), miR160h (0,91) |

**Supplementary Table 3** Hepatic gene regulation in mice. Genes modulated by the *mol*-miR pool reported by function, considering the QIAGEN gene list for Adipogenesis and Fatty Liver. The fold changes (FC) of the upregulated genes are reported in bold; the FC of the downregulated genes are reported in italic

| **Ref. Seq.** | **Gene** | | **ND+*mol‑*miRs *vs*  ND** | | **HFD  *vs*  ND** | | **HFD+*mol‑*miRs  *vs*  HFD** | | **HFD+*mol‑*miRs *vs*  ND** |
| --- | --- | --- | --- | --- | --- | --- | --- | --- | --- |
| Regulation of Adipogenesis | | | | | | | | | |
| Adipokines | | | | | | | | | |
| NM_009605 | Adipoq | | **2,6123** | | 6,7225 | | *0,3577* | | **2,4049** |
| NM_013459 | Cfd | | 1,0551 | | **12,9511** | | *0,1466* | | 1,8987 |
| NM_008493 | Lep | | 0,8738 | | **55,8699** | | *0,0338* | | 1,8895 |
| NM_022984 | Retn | | 1,5035 | | **66,7641** | | *0,0143* | | 0,9526 |
| Hormones | | | | | | | | | |
| NM_007428 | Agt | | 1,4293 | | **6,9692** | | 0,5274 | | **3,6757** |
| Lipases | |  | |  | |  | |  | |
| NM_008509 | Lpl | | **2,4105** | | **4,1785** | | *0,4681* | | 1,9561 |
| Pro-Adipogenesis | | | | | | | | | |
| NM_007679 | Cebpd | | 1,9444 | | **26,1547** | | *0,0584* | | 1,5273 |
| NM_010051 | Dkk1 | | 1,8952 | | **84,098** | | *0,0406* | | **3,4105** |
| NM_007891 | E2f1 | | 1,2306 | | **3,8718** | | *0,3716* | | 1,4389 |
| NM_024406 | Fabp4 | | 1,7223 | | *0,3475* | | **2,7549** | | 0,9573 |
| NM_013519 | Fgf2 | | 0,8187 | | **24,8298** | | *0,0297* | | 0,7366 |
| NM_013834 | Sfrp1 | | 1,9123 | | **36,6566** | | *0,0755* | | **2,7664** |
| NM_018780 | Sfrp5 | | **2,2444** | | **66,7641** | | *0,039* | | **2,6063** |
| Anti-Adipogenesis | | | | | | | | | |
| NM_010052 | Dlk1 | | **3,905** | | **112,9077** | | *0,0311* | | **3,5137** |
| NM_009822 | Runx1t1 | | 0,6922 | | **23,7359** | | *0,0795* | | 1,8882 |
| Pro-White Adipose Tissue | | | | | | | | | |
| NM_010118 | Egr2 | | **4,2115** | | **14,97** | | *0,1266* | | 1,8947 |
| NM_008006 | Fgf10 | | 1,8691 | | **77,6546** | | *0,0217* | | 1,6818 |
| NM_010637 | Klf4 | | 1,889 | | **14,2907** | | *0,0891* | | 1,2728 |
| Anti-White Adipose Tissue | | | | | | | | | |
| NM_008090 | Gata2 | | **2,0368** | | **52,3457** | | *0,035* | | 1,8327 |
| NM_008091 | Gata3 | | 0,567 | | **44,2008** | | *0,0231* | | 1,0203 |
| NM_008452 | Klf2 | | **2,215** | | **38,4524** | | *0,0328* | | 1,2605 |
| Pro-Brown Adipose Tissue | | | | | | | | | |
| NM_010050 | Dio2 | | 0,8823 | | **47,5048** | | *0,0167* | | 0,7939 |
| NM_008090 | Foxc2 | | 1,0772 | | *0,0819* | | 11,8351 | | 0,9693 |
| NM_011951 | Mapk14 | | **2,3955** | | **3,8504** | | *0,2774* | | 1,0681 |
| NM_010938 | Nrf1 | | 1,5826 | | **3,7659** | | *0,4659* | | 1,7544 |
| NM_009463 | Ucp1 | | 0,8835 | | *0,0992* | | **17,3276** | | 1,7195 |
| Anti-Brown Adipose Tissue | | | | | | | | | |
| NM_009029 | Rb1 | | 1,8498 | | **6,3467** | | *0,355* | | **2,2532** |
| Metabolic Pathways | |  | |  | |  | |  | |
| Beta-Oxidation | | | | | | | | | |
| NM_010570 | Irs1 | | 1,2669 | | *0,3989* | | **7,1801** | | **2,8639** |
| NM_011144 | Ppara | | 0,8013 | | **2,3784** | | *0,2655* | | 0,6316 |
| Cholesterol Metabolism & Transport | | | | | | | | | |
| NM_011145 | Ppard | | 0,6477 | | **3,5578** | | *0,3281* | | 1,1672 |
| NM_011305 | Rxra | | 0,9287 | | **7,3973** | | *0,1665* | | 1,232 |
| Tumorigenesis | | | | | | | | | |
| NM_009733 | Axin1 | | 1,1434 | | *0,2672* | | **2,6226** | | 0,7008 |
| NM_007553 | Bmp2 | | 1,4136 | | **27,7228** | | *0,0459* | | 1,2719 |
| NM_007557 | Bmp7 | | 1,3098 | | **6,9596** | | *0,1693* | | 1,1785 |
| NM_009870 | Cdk4 | | 1,0668 | | **2,9019** | | *0,2936* | | 0,852 |
| NM_007669 | Cdkn1a | | 1,9084 | | **8,0724** | | *0,2657* | | **2,145** |
| NM_009170 | Shh | | *0,4973* | | **11,0655** | | *0,1472* | | 1,629 |
| NM_009271 | Src | | 0,7097 | | **5,3369** | | *0,3244* | | 1,7315 |
| NM_011658 | Twist1 | | 0,93 | | **4,1728** | | *0,4081* | | 1,7029 |
| NM_009504 | Vdr | | 1,2873 | | **61,7343** | | *0,0239* | | 1,4763 |
| NM_009522 | Wnt3a | | **2,3757** | | **47,4061** | | *0,0329* | | 1,5583 |
| NM_009524 | Wnt5a | | 1,2187 | | **12,295** | | *0,1222* | | 1,5021 |
| NM_011718 | Wnt10b | | 1,643 | | **39,7257** | | *0,062* | | **2,464** |

**Supplementary Table 4** Genes modulated by MOES in the HepG2 cell line and by *mol*-miR pool in pre-obese mice The fold changes (FC) of the upregulated genes are reported in bold; the FC of the downregulated genes are reported in italic

| **Gene name** | **HFD *vs* ND** | **HFD + *mol‑*miR *vs* ND** | **HepG2 + MOES *vs* HepG2** | ***mol*-miR target human genes** | ***mol*-miR target  murine genes** |
| --- | --- | --- | --- | --- | --- |
| Agt | 6,9692 | 3,6757 | *0,4739* | miR160h (0.65), miR395d (0.96), miR482b (0.97) | miR160h(0,90), miR159c(0,90), miR167f-3p(0,96), miR397a(0,68) |
| Cdkn1a | 8,0724 | 2,145 | *0,4401* | miR395d (0.93), miR160h (0.96), miR166 (0.97) | miR160h(0,96) |
| Cfd | 12,9511 | 1,8987 | *0,2981* | miR160h (0.95) | miR160h(0,97) |
| Dio2 | 47,5048 | 0,7939 | 2,3686 | miR166 (0.96), miR396a (0.99) | miR159c(0,94), miR156e(0,94), miR166(0,87), |
| Egr2 | 14,97 | 1,8947 | *0,0089* | miR166 (0.60), miR159c (0.82) | miR167f-3p(0,95), miR159c(0,94) |
| Insr | 4,9041 | 2,5193 | *0,3864* | miR396a (0.85), miR482b (0.98) | miR395d(0,90) |
| Klf2 | 38,4524 | 1,2605 | *0,4797* | miR2118a (0.87), miR482b (0.96), miR166 (0.97) | miR166(0,97), miR160h(0,88) |
| Lep | 55,8699 | 1,8895 | 3,7469 | miR159c (0.95), miR482b (0.96), miR160h (0.96), miR166 (0.98) | miR395d(0,95), miR159c(0,91), miR160h(0,92) |
| Lpl | 4,1785 | 1,9561 | *0,2644* | miR160h (0.61) | miR167f-3p(0,65), |
| Mapk14 | 3,8504 | 1,0681 | *0,1788* | miR393a (0.92), miR396a (0.98) | miR159c(0,93) |
| Nrf1 | 3,7659 | 1,7544 | *0,2898* | miR160h (0.94), miR396a (0.97), miR482b (0.98) | miR167f-3p(0,85) |
| Ppara | 2,3784 | 0,6316 | *0,0448* | miR393a (0.75), miR160h (0.96), miR166 (0.96) | miR397a(0,96), miR482b(0,94), miR160h(0,87) |
| Rb1 | 6,3467 | 2,2532 | *0,1757* | miR160h (0.86) | miR159c(0,61) |
| Rxra | 7,3973 | 1,232 | *0,0834* | miR160h (0.97), miR166 (0.98) | miR160h(0,93), miR159c(0,93), miR398a-5p(0,61), miR166(0,61) |
| Sfrp1 | 36,6566 | 2,7664 | *0,169* | miR160h (0.84), miR166 (0.84), miR482b (0.97) | miR160h(0,92), miR166(0,92), |
| Shh | 11,0655 | 1,629 | *0,143* | miR159c (0.95), miR482b (0.98), | miR858b(0,96), miR167f-3p(0,72) |
| Vdr | 61,7343 | 1,4763 | *0,2951* | miR160h (0.71), miR166 (0.97) | miR160h(0,91), miR398a-5p(0,91), miR166(0,63) |
| Wnt3a | 47,4061 | 1,5583 | *0,0139* | miR171b (0.87), miR166 (0.92), miR482b (0.94), miR2118a (0.96), miR160h (0.98) | miR159c(0,93), miR395d(0,85) |
| Wnt10b | 39,7257 | 2,464 | *0,167* | miR166 (0.62), miR167f-3p (0.88), miR395d (0.96), miR482b (0.96), miR2118a (0.96), miR160h (0.98) | miR858b(0,96), miR167f-3p(0,94), miR160h(0,91) |
